# Exon-centric regulation of ATM expression is population-dependent and amenable to antisense modification by pseudoexon targeting

**DOI:** 10.1038/srep18741

**Published:** 2016-01-06

**Authors:** Jana Kralovicova, Marcin Knut, Nicholas C. P. Cross, Igor Vorechovsky

**Affiliations:** 1 University of Southampton Faculty of Medicine Southampton SO16 6YD United Kingdom; 2 Wessex Regional Genetics Laboratory Salisbury Hospital Salisbury SP2 8BJ United Kingdom

## Abstract

*ATM* is an important cancer susceptibility gene that encodes a critical apical kinase of the DNA damage response (DDR) pathway. We show that a key nonsense-mediated RNA decay switch exon (NSE) in *ATM* is repressed by U2AF, PUF60 and hnRNPA1. The NSE activation was haplotype-specific and was most promoted by cytosine at rs609621 in the NSE 3′ splice-site (3′ss), which is predominant in high cancer risk populations. NSE levels were deregulated in leukemias and were influenced by the identity of U2AF35 residue 34. We also identify splice-switching oligonucleotides (SSOs) that exploit competition of adjacent pseudoexons to modulate NSE levels. The U2AF-regulated exon usage in the ATM signalling pathway was centred on the MRN/ATM-CHEK2-CDC25-cdc2/cyclin-B axis and preferentially involved transcripts implicated in cancer-associated gene fusions and chromosomal translocations. These results reveal important links between 3′ss control and ATM-dependent responses to double-strand DNA breaks, demonstrate functional plasticity of intronic variants and illustrate versatility of intronic SSOs that target pseudo-3′ss to modify gene expression.

Introns are removed by a large and highly dynamic RNA-protein complex termed the spliceosome, which orchestrates complex interactions between primary transcripts, small nuclear RNAs (snRNAs) and a large number of proteins^1^. Spliceosomes assemble *ad hoc* on each intron in an ordered manner, starting with recognition of the 5‘ splice site (5’ss) by U1 snRNA or the 3′ss by the U2 pathway^1^,^2^, which involves binding of the U2 auxiliary factor (U2AF) to the 3′ss region to facilitate U2 recognition of the branch point sequence (BPS)^3^. U2AF is a stable heterodimer composed of a *U2AF2*-encoded 65-kD subunit (U2AF65), which binds the polypyrimidine tract (PPT), and a *U2AF1*-encoded 35-kD subunit (U2AF35), which interacts with highly conserved AG dinucleotides at 3′ss and stabilizes U2AF65 binding^4^. In addition to the BPS/PPT unit and 3′ss/5′ss, accurate splicing requires auxiliary sequences or structures that activate or repress splice site recognition, known as intronic or exonic splicing enhancers or silencers. These elements allow genuine splice sites to be recognized among a vast excess of cryptic or pseudo-sites in the genome of higher eukaryotes, which have similar sequences but outnumber authentic sites by an order of magnitude^5^. Although they often have a regulatory function^6^, molecular mechanisms of their repression are poorly understood.

Exome sequencing studies have revealed a restricted pattern of somatic mutations in *U2AF1/U2AF2* and other genes involved in 3′ss recognition in cancer cells, including *SF3B1, ZRSR2, SF1, SF3A1, PRPF40B,* and *SRSF2* (reviewed in^7^). These genes encode products that often interact during spliceosome assembly^8^,^9^,^10^ and exhibit a high degree of mutual exclusivity of cancer-associated mutations^7^, pointing to the existence of a shared oncogenic pathway. Transcriptome profiling in leukemias carrying these mutations detected numerous alterations in splicing of mRNA precursors^7^, but key links between specific RNA processing defects and cancer initiation or progression have remained obscure, despite the great promise of these targets for therapeutic modulation. In addition, it has been unclear why the highly restricted mutation pattern in these cells has not been associated with a limited and clearly defined set of RNA processing defects with oncogenic properties. Furthermore, exon usage in DDR genes, critical players in malignant transformation, has not been fully characterized in cells lacking 3′ss processing factors and natural DNA variants that influence their activation have been unknown.

Here, we identify a U2AF-repressed nonsense-mediated decay (NMD) switch exon in *ATM* (ataxia-telangiectasia, A-T, mutated). We show that the extent to which this event limits ATM expression depends largely on a common intronic variant rs609261 located in the NSE 3′ss. By exploiting novel intronic *cis*-elements that control NSE inclusion levels, we identify SSOs that modulate NSE activation by targeting a competing pseudoexon in the same intron. In addition, we identify U2AF-related proteins that control NSE selection. Using RNA-Seq, we also show that the U2AF-mediated regulation of DDR pathways is centred on the ATM-CHEK2-CDC25-cdc2/cyclin-B axis, suggesting that it has coevolved with cellular responses to double-strand DNA breaks (DSBs). Finally, we demonstrate a preferential involvement of U2AF-regulated transcripts in cancer-associated gene fusions and chromosome translocations.

## Results

### Identification of a U2AF-repressed cryptic exon in *ATM*

**Figure 1:**
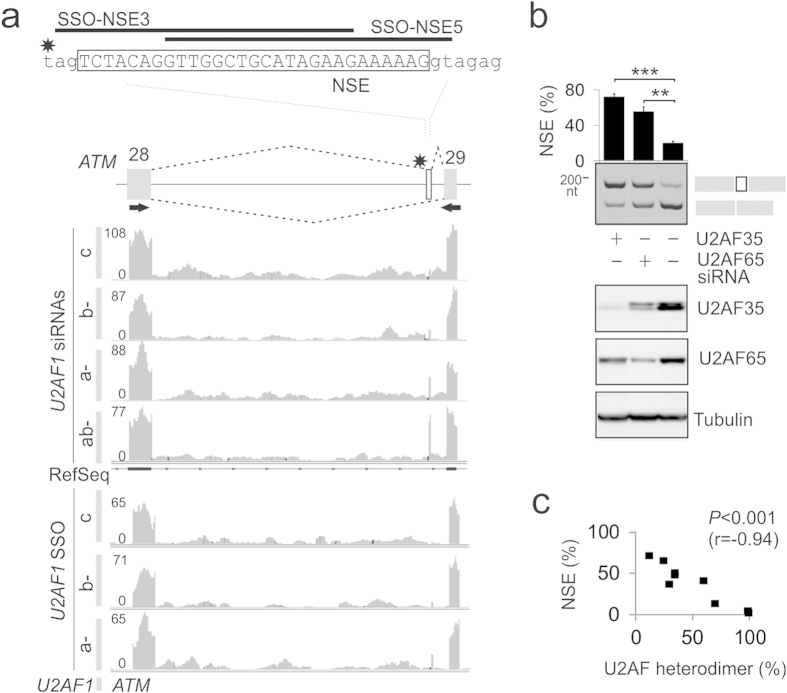
Identification of a U2AF-repressed cryptic exon in *ATM* intron 28. (a) Schematics of NSE activation. NSE sequence (*upper panel*) is boxed, asterisk denotes rs609621, black rectangles show the indicated antisense oligonucleotides. Genome browser views of RNA-Seq data from RNAi- or SSO-mediated depletions of both U2AF35 isoforms (ab-), U2AF35a (a-), U2AF35b (b-) and controls (c) are shown in the *lower panel*. SSOs targeting 3′ ss of *U2AF1* exons Ab and 3 and U2AF35 siRNAs were as described^11^. Y axis, read densities. NSE inclusion/exclusion is schematically shown by dotted lines at the top. *ATM* exons (gray boxes) are numbered as in ref. 13. The 29-nt NSE introduced a stop codon in the *ATM* mRNA. (**b)** Validation of the NSE activation by RT-PCR (*upper panel*) in independent depletions (*lower panel).* RT-PCR primers (ATM-F, ATM-R, Table S1) are denoted by arrows in panel **a**. Spliced products are shown to the right, the percentage of transcripts with NSE is at the top. Error bars denote SDs of two transfections experiments. ***p < 0.0001, **p < 0.001. **(c)** NSE inclusion in mature transcripts inversely correlates with residual U2AF. r, Pearson correlation. Estimates of heterodimer levels were as described^11^.

We and others have recently shown that depletion of each U2AF subunit resulted in down- and up-regulation of a large number of exons that were predominantly alternatively spliced^11^,^12^. When inspecting global RNA processing changes in cells depleted of U2AF35, we found an unexpectedly strong activation of a cryptic, 29-nt *ATM* exon that was not annotated by RefSeq (termed NSE for NMD switch exon, Fig. 1a). The NSE activation was observed also in cells individually depleted of each U2AF35 isoform with isoform-specific small interfering RNAs (siRNAs) and with SSOs targeting 3′ss of alternatively spliced *U2AF1* exons Ab and 3, which encode isoform U2AF35b and U2AF35a, respectively (Fig. 1a). Validation of RNA-Seq data using RT-PCR showed that NSE was present in ~10-20% of polyadenylated transcripts in untreated human embryonic kidney (HEK) 293 cells, similar to levels observed in lymphoblastoid cell lines^13^. The NSE inclusion levels increased to ~75% in cultures depleted of ~90% U2AF35 and to ~50% in cells depleted of ~75% U2AF65 (Fig. 1b), were siRNA dose-dependent and inversely correlated with the estimated amount of available U2AF heterodimers (Fig. 1c), consistent with the requirement of each U2AF subunit for NSE repression. RNA-Seq data also revealed retention of intronic sequences surrounding NSE (Fig. 1a) but not adjacent introns, suggesting that intron 28 is ‘detained’ and could be spliced post-transcriptionally^14^. Retention levels of intron 28 were affected neither by SSO- nor siRNA-mediated depletion of U2AF35 (Fig. 1a) and no other cryptic exon in this gene was activated to the same extent as NSE. Thus, NSE plays an important role in the exon-centric regulation of *ATM* expression by U2AF.

### NSE activation and ATM expression is modified by rs609261

**Figure 2:**
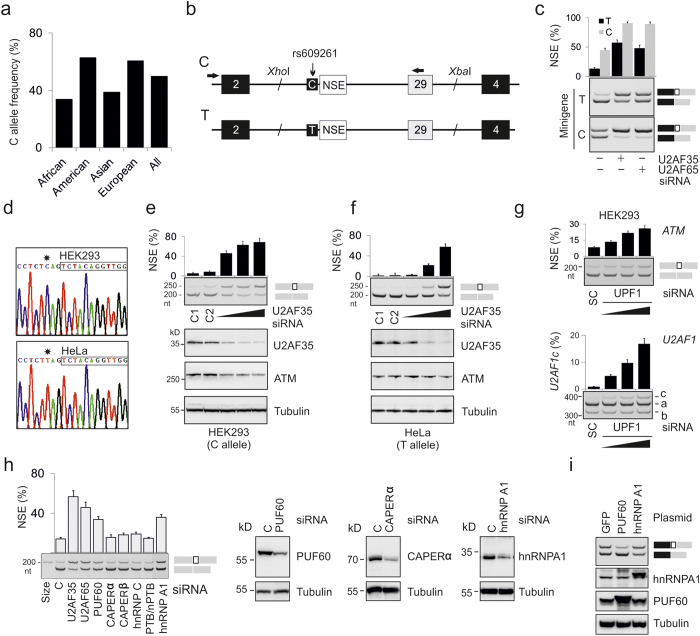
**NSE activation and ATM expression are modified by rs609261. (a) Allelic frequencies at rs609261 in the indicated populations**^66^. (**b**) Minigene schematics. An *Xho*I/*Xba*I segment of *ATM* containing NSE and exon 29 was cloned between *U2AF1* exons 2 and 4 (*black boxes*). RT-PCR primers to amplify exogenous transcripts (PL3 and ATM-R, Table S1) are denoted by arrows. (**c**) The rs609261-dependent NSE activation in exogenous pre-mRNAs. HEK293 cells depleted of U2AF35 or U2AF65 were transiently transfected with T (*black*) and C (*gray*) minigenes. Final concentration of the U2AF35 and U2AF65 siRNAs was 30 and 60 nM, respectively. (**d**) Identification of cell lines homozygous at rs609261 (*asterisk*). NSE is boxed. (**e,f**) Allele-specific activation of NSE in endogenous transcripts limits ATM expression in a dose-dependent manner. The source of endogenous transcripts is at the bottom, antibodies are to the right. Concentration of siRNAs in HEK293 cultures was 3, 10 and 30 nM. Concentration of siRNAs in HeLa cultures was 6.6, 20 and 60 nM. C1, C2, control siRNAs. Transfection efficiency was monitored by a GFP-plasmid and fluorescent microscopy. (**g**) UPF1 depletion increased NSE activation (*upper panel*) and upregulated isoform *U2AF1c (lower panel)*. The *U2AF1c* isoform contains both exons Ab and 3 and is repressed by NMD^11^,^67^. Final concentration of the UPF1 siRNA was 7, 20 and 60 nM. SC, a scrambled control. Error bars are SDs of independent transfections. (**h**) NSE inclusion levels in cells depleted of U2AF-related proteins and a subset of heterogenous nuclear RNPs. Error bars denote SDs of two transfections. Immunoblots are shown to the right. Final concentration of the U2AF35 siRNA was 25 nM; the remaining siRNAs were at 60 nM. C, controls. (**i**) Overexpression of PUF60 induced NSE skipping. Immunoblots are shown below, antibodies to the right.

Examination of genomic sequences surrounding NSE revealed that position -3 relative to the NSE 3′ss is polymorphic at rs609261 where thymine (T) is predominant in African and Asian populations and cytosine (C) in Caucasians (Fig. 2a). The base identity at this position is critical for universal exon recognition, with a CAG>TAG>AAG>GAG hierarchy of exon inclusion levels at the 3′ss consensus^15^. To confirm that the NSE usage is allele-specific, we examined splicing of two reporter constructs that contained C or T at this position following transient transfections into HEK293 cells (Fig. 2b). As expected, the T construct yielded lower NSE inclusion than the C reporter, both in untreated cells and cells individually depleted of each U2AF subunit (Fig. 2c).

To test whether the allele-specific NSE usage results in differential protein expression in cells lacking U2AF35, we first sequenced DNA from available cell lines across rs609621 to obtain transfectable cells homozygous for each allele. We found that HEK293 cells were homozygous for the C allele and HeLa cells were homozygous for the T allele (Fig. 2d). Immunoblots from the U2AF35-depleted cells and untreated controls confirmed efficient depletion in each cell line and a greater U2AF-mediated decline of ATM expression in the presence of the C allele than the T allele (Fig. 2e,f and Fig. S1a–c). Depletion of UPF1, a key component of the NMD pathway, revealed a dose-dependent increase of NSE inclusion in *ATM* mRNAs (Fig. 2g). No signal from a putative truncated ATM was detected on immunoblots from depleted cells.

Because U2AF-repressed and -activated exons show preferential responses to U2AF-related proteins^11^, we depleted HEK293 cells of PUF60 and CAPERα, and also several heterogeneous nuclear ribonucleoproteins, including hnRNP A1. PUF60 interacts with uridine-rich motifs at 3′ss^16^ and hnRNP A1 forms a ternary complex with U2AF on AG-containing U-rich RNAs^17^. Depletion of either PUF60 or hnRNP A1 increased NSE inclusion (Fig. 2h) while PUF60 overexpression led to NSE skipping (Fig. 2i). Together, these results show that the rs609261- and population-dependent NSE activation deep in *ATM* intron 28 is regulated by U2AF, PUF60 and hnRNP A1 and demonstrate that functionality of a common intronic polymorphism varies with cellular levels of RNA-binding proteins that facilitate 3′ss recognition.

### NSE inhibition by SSOs promotes ATM expression

**Figure 3:**
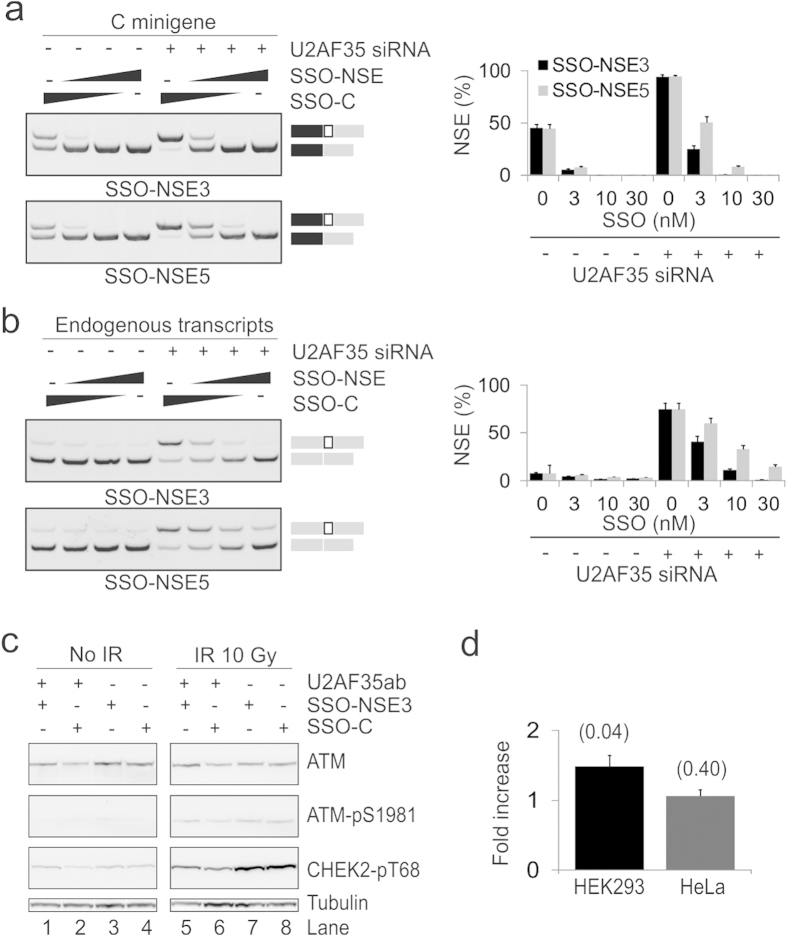
Rescue of U2AF-repressed ATM expression by SSOs targeting NSE. (a,b) Efficient SSO-mediated NSE inhibition in exogeneous (a) and endogenous (b) AT*M* transcripts. Mean NSE inclusion levels of two transfection experiments are shown in the *right panels*. (**c**) Restoration of ATM protein levels by SSOs that blocks access to NSE. Cells lacking U2AF35 and control cells were transfected with the SSO targeting the NSE 3’ss and a control SSOs (Fig. 1a and Table S1), as described^61^,^68^. After 48 hrs, the cells were exposed to ionizing radiation (IR, 10 Gy) and harvested 1 hr later. Cell lysates were separated using a gradient SDS-PAGE. Western blotting was with antibodies shown to the right. (**d**) SSO-NSE3-mediated enhancement of ATM protein in cell lines homozygous at rs609261. Columns represent the average fold difference in ATM expression between SSO-NSE3-treated and SSO-C-treated homozygous cells depleted of U2AF35. Error bars represent SD of two biological replicates. P-values comparing SSO-C with SSO-NSE3-treated counterparts are shown at the top. Representative immunoblots are in Fig. S1a,b.

To test if NSE activation in cells lacking U2AF can be repressed to restore ATM expression, we first individually cotransfected the C-allele reporter construct with SSOs targeting NSE splice sites (Fig. 1a). SSOs were modified with phosphorothioate linkages at each end and with 2′-O-methyl at each ribose and were designed to avoid the PPT of NSE, stable Mfold-predicted stems and rs609261. Each SSO diminished NSE inclusion in a dose-dependent manner both in exogeneous (Fig. 3a) and endogeneous (Fig. 3b) transcripts and the SSO targeting the NSE 3′ss was more efficient than the SSO bridging its 5’ss at the same concentrations.

We next examined if the NSE 3′ss SSO can increase ATM protein expression. The reduced ATM expression in cells lacking U2AF35 was partially rescued by this SSO in HEK293 cells but this was not apparent in HeLa cells where the NSE activation and reconstitution was less efficient at the same SSO concentrations (Fig. S1a and S1b, lanes 5–8 vs 9–12; Fig. S1c). We also tested if the SSO NSE3 can influence downstream ATM targets in HEK293 cells exposed to ionizing radiation (IR). The low ATM expression in cells lacking U2AF35 was partially rescued by this SSO also in IR-exposed cells (Fig. 3c, lanes 1 vs 2 and 5 vs 6, and Fig. S1e, lanes 5–8 vs 9–12). Following IR, activated ATM autophosphorylated at S1981^18^ was reduced in depleted cells as compared to untreated cells (Fig. S1e, lanes 1–4 vs 5–8 and Fig. 3c, lane 6 vs 8) and this signal also appeared slightly increased following exposure to the NSE 3′ss SSO (Fig. S1e, lanes 5–8 vs 9–12, and Fig. 3c, lane 5 vs 6). We also overexpressed wild-type CHEK2 in (mock)-irradiated cells (mock)-depleted of U2AF, a serine/threonine kinase phosphorylated by ATM at T68 in response to DSBs^19^. Cells lacking U2AF had markedly lower levels of endogenous CHEK2 compared to controls, which did not appear to be altered by the NSE 3′ss SSO (Fig. S1e, lanes 1–12). In contrast, exogeneous CHEK2 was increased in depleted cells both in IR-exposed and -unexposed cells (lanes 1–4 vs 5–8, see also Fig. S1f,g further below).

Taken together, NSE activation was efficiently inhibited by SSOs that block access to NSE splice sites and do not support RNase H cleavage. The more efficient SSO partially rescued the NSE-mediated inhibition of ATM.

### NSE activation is regulated by a downstream pseudoexon

**Figure 4:**
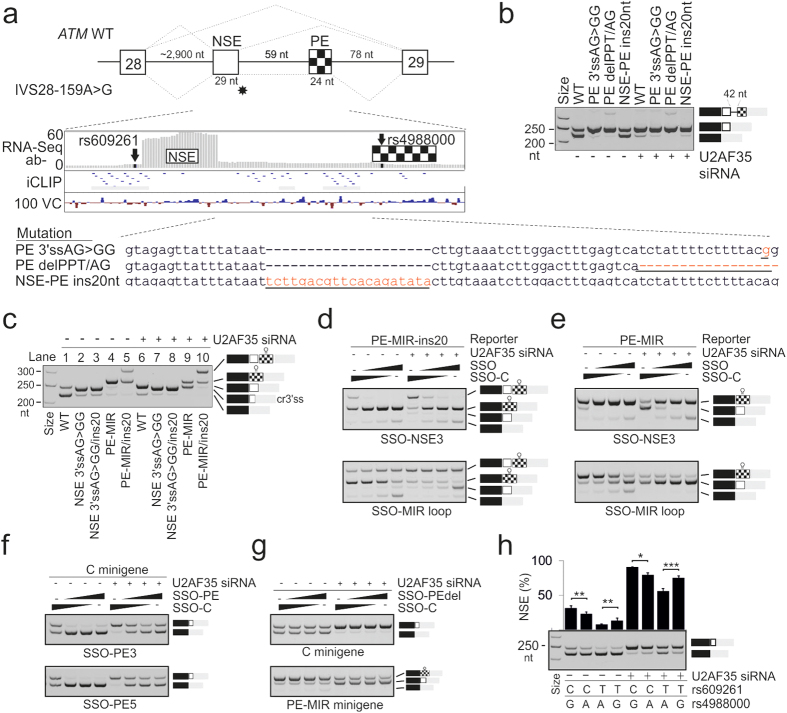
Identification of intronic *cis*-elements and SSOs that modulate NSE activation. (a) Schematics of two pseudoexons in *ATM* intron 28. Canonical exons, NSE and PE are shown as gray, white and checkered boxes, respectively. Asterisk indicates location of the IVS28-159A >G substitution^13^. In this A-T case, both NSE and PE were included in the *ATM* mRNA together with the intervening sequence. Canonical and aberrant transcripts are denoted by dotted lines above and below the pre-mRNA. *Middle panel* shows RNA-Seq read densities for NSE in cells depleted of both U2AF35 isoforms (ab-) together with U2AF65 tags/high-confidence binding sites (horizontal lines/rectangles) identified by crosslinking and immunoprecipitation^20^. The 100 basewise vertebrate conservation by Phylop (100 VC) is at the bottom. The *lower panel* shows mutations (*in red and underlined*) introduced in the C-minigene. (**b**) Splicing pattern of wildtype and mutated C minigenes. Mutations are at the bottom of panel (**a**); RNA products are shown schematically to the right. The largest product produced by clone PE delPPT/AG includes the shortened pseudointron. (**c**) Splicing pattern of C minigenes mutated in NSE (lanes 2, 3, 7 and 8) or PE (lanes 4, 5, 9 and 10) in (mock)-depleted HEK293 cells. Mutations are at the bottom; their sequences are in Table S2. Spliced products are schematically shown to the right. A hairpin symbol above PE denotes the MIR stem-loop insertion; cr3’ss, a cryptic 3’ss activation 7 nt downstream of the authentic NSE 3’ss. (**d,e**) SSO-induced pseudoexon switching. Transfected minigenes are shown at the top, spliced products to the right and SSOs at the bottom. SSO sequences are in Table S1. Final concentration of SSOs shown in panels (**d–g**) was 3, 10 and 30 nM. (**f**) PE SSOs induced NSE skipping. (**g**) SSOs targeting a sequence that activates NSE upon deletion (PEdelPPT/AG; panel **a** and **b**) inhibit PE. (**h**) NSE activation is haplotype-dependent. Minigene haplotypes at the indicated variants are at the bottom. Columns represent mean NSE inclusion, error bars are SDs, asterisks denote statistically significant differences as in Fig. 1b.

To identify intronic regulatory *cis*-elements that control NSE inclusion in mature transcripts, we took advantage of a previously reported A-T mutation IVS28-159A >G^13^. We noticed that this mutation activated the NSE 3′ss while repressing its 5’ss and promoting a downstream 5’ss instead, introducing a 112-nt cryptic exon in the mRNA^13^. We also noticed that within this exon, there was a strong 3′ss consensus preceded by optimal BPS/PPT motifs, which may bind U2AF and activate a smaller, 24-nt pseudoexon (termed PE; Fig. 4a). Examination of published RNA crosslinking/immunoprecipitation data^20^ in *ATM* showed U2AF65 binding upstream and downstream of NSE and upstream of PE, suggesting that NSE activation may be controlled by competition between partially productive spliceosomes assembled at the PE 3′ss and the NSE 3′ss. The two 3′ss are conserved in mammals and are separated by a distance smaller than the minimal intron size, sterically preventing simultaneous recognition of NSE and PE (Fig. 4a). Deletion of the PE PPT/3′ss introduced in the C minigene, which should alleviate NSE repression through diminished U2AF binding to PE, increased NSE inclusion (Fig. 4b), in agreement with the 3′ss competition. This deletion also induced retention of the intron that separates NSE and PE, mimicking the splicing pattern of the A-T mutation IVS28-159A >G. Increasing the intron length from 59 to 79 nt, thereby overcoming a steric hindrance imposed by the insufficient distance between the two pseudo-3′ss, also improved NSE inclusion and diminished the intron retention (Fig. 4b).

To test if NSE can influence PE inclusion in the mRNA, we first eliminated the NSE 3′ss. The NSE 3′ss mutation activated a cryptic 3′ss 7-nt downstream, which had a diminished requirement for U2AF (Fig. 4c, lanes 1, 2 and 6, 7, Table S2). Because extending the intron between NSE and PE on this background also failed to activate PE (lanes 3 and 8) and because PE lacks exonic splicing enhancers and has a suboptimal BPS (Table S3), we inserted a 24-nt stemloop derived from a mammalian-wide interspersed repeat (MIR) in the middle of PE. This MIR hairpin acts as a nearly universal exon definition module through an exposed splicing enhancer in a terminal RNA triloop^21^. The enlarged PE was strongly activated in mock-depleted cells, but was outcompeted by NSE in cells lacking U2AF35 (lanes 4 and 9), demonstrating differential requirements of the two pseudoexons for U2AF(35). The construct containing both the MIR insertion in PE and the extended intron finally generated mRNAs containing both NSE and PE (lanes 5 and 10).

### Intronic SSOs targeting competing pseudoexons to modulate gene expression

Next, we employed this MIR reporter to test the impact of NSE and PE SSOs on exon usage and ATM expression. Figure 4d shows that the NSE 3′ss SSO repressed transcripts containing NSE and upregulated those with PE whereas the opposite effect was found for SSOs targeting the MIR enhancer loop in PE. We observed the same pattern for the reporter in which NSE and PE were separated by a distance insufficient for their simultaneous inclusion in the mRNA (Fig. 4e). These results suggested that SSOs targeting PE and/or U2AF65 binding sites upstream of PE may potentially promote NSE inclusion and reduce ATM expression, cancelling out the NSE SSOs. This approach could provide a broader strategy for intronic SSOs to modulate gene expression in either direction by targeting competing pseudoexons, one of which is critical for gene regulation. To test this concept, we examined PE SSOs. Although SSOs targeting PE splice sites induced NSE skipping in exogeneous and endogeneous transcripts (Fig. 4f), SSOs blocking access to U2AF65 binding sites just upstream of PE (Fig. 4a), ie. to the NSE-repressing motif identified in construct delPPT/AG (Fig. 4b), reduced PE inclusion and slightly increased NSE in the MIR reporter (Fig. 4g). A longer oligo extending in the 5’ direction (SSO-PEBP, Table S1) did not alter NSE inclusion levels.

PE contains a natural G/A variant rs4988000, which may also influence NSE recognition (Fig. 4h). Transfections of C and T minigenes systematically mutated at rs4988000 revealed that the rare A allele at rs4988000 decreased NSE inclusion on each pre-mRNA, both in U2AF35- and mock-depleted cells. Thus, the highest NSE inclusion was produced by the most frequent haplotype in Caucasians (CG), followed by haplotypes CA >TG > TA.

Taken together, the haplotype-dependent activation of the U2AF-repressed NSE can be modified by SSOs that target splicing regulatory motifs of competing pseudo-3′ss.

### Regulation of ATM signalling by U2AF: DSBs at the focal point

**Figure 5:**
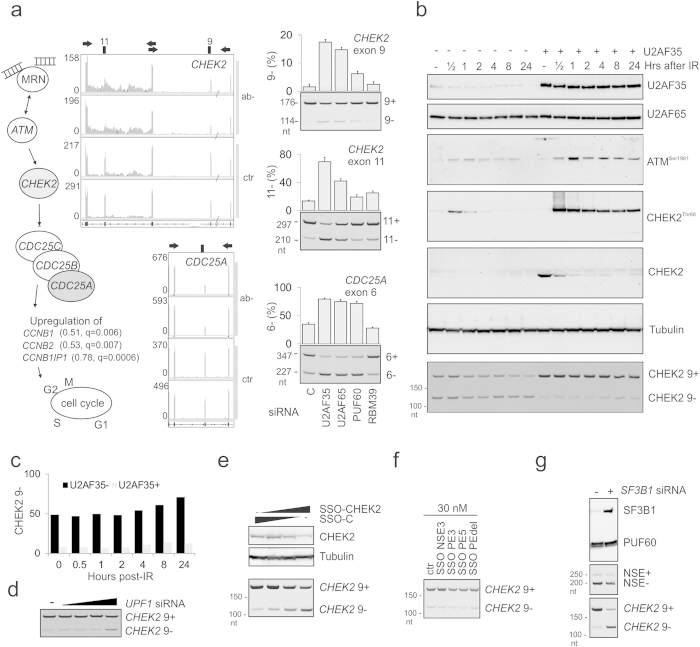
Exon-centric regulation of ATM signalling. (**a**) U2AF-regulated gene- and exon-level expression changes in MRN-ATM-CHEK2-CDC25-cdc2/cyclin B pathway (*left panel*). Log2fold- and q-values^63^ are shown in parentheses. Exon usage of *CHEK2* and *CDC25A* genes is shown by RNA-Seq browser shots; PCR validation gels are in the *right panels*. *CHEK2* exon 9 is a NMD switch exon; exon 11 encodes a portion of the kinase domain. Full spectrum of U2AF-mediated expression changes in the ATM signalling pathway is shown in Fig. S2; examples of the U2AF-mediated splicing regulation are in Figs S3–S6. (**b**) Impaired ATM signalling in U2AF35 depleted cells following IR. HEK293 cells were (mock)depleted of U2AF35 and subjected to IR (10 Gy) 48 hrs later. Expression was examined by immunoblotting at the indicated time points. Antibodies are shown to the right. *CHEK2* exon 9 skipping levels are at the bottom; their measurements in control (U2AF35+) and depleted cells (U2AF35−) are in panel (**c**). (**d**) C*HEK2* exon 9 inclusion in UPF1 depleted cells. Final concentration of the *UPF1* siRNA (Table S1) was 12.5, 25, 50, and 100 nM. (**e**) Repression of *CHEK2* exon 9 by SSO reduced CHEK2 levels and promoted NSE inclusion. Final concentration of SSO targeting *CHEK2* exon 9 was 3, 10 and 30 nM. (**f**) *CHEK2* exon 9 inclusion upon transfection of HEK293 cells with the indicated SSOs. (**g**) A lack of SF3B1 induced *CHEK2* exon 9 skipping but did not alter NSE activation. Final concentration of each siRNA targeting *SF3B1* was 20 nM.

Because ATM is a key apical kinase in the DDR pathway^22^ and NMD switch exons altered by U2AF were frequent in genes encoding U2AF protein interaction partners^11^, we systematically characterized U2AF(35)-induced RNA processing changes of currently known ATM substrates^23^ and other constituents of the ATM signalling network^24^. Interestingly, although genes involved in the DDR and cell cycle control that contained U2AF(35)-dependent exons were only marginally enriched (FDR = 0.08)^11^, each component in the ATM-CHEK2-CDC25-CDC2/cyclin B axis showed RNA processing alterations (Fig. 5a, Fig. S2). This pathway is critical for ATM signalling of DSBs^22^.

First, reduced ATM expression in cells lacking U2AF (Fig. S1e) was associated with decreased *CHEK2* mRNA, increased retention of *CHEK2* intron 10, and skipping of exons 9 and 11 (Fig. 5a). RNA processing alterations of known CHEK2 substrates were limited to genes regulating the cell cycle (*CDC25A, CDC25B, CDC25C* and *TTK;* Fig. 5a, S3a,b, S4a) and were not apparent in genes involved in DNA repair (*BRCA1/2, XRCC1, FOXM1, TRIM28*) or p53 signalling (*TP53, MDM4, CABIN1, STRAP, AATF*). *CHEK2* exon 9 skipping, which is predicted to activate NMD, was only marginally increased 24 hrs after IR and did not appear to contribute to the decline of total CHEK2 observed as early as 30 min after IR (Fig. 5b,c). As *CHEK2* exon 9 inclusion was increased only for the highest concentration of tested *UPF1* siRNAs (Fig. 5d), we transfected HEK293 cells with an SSO targeting its 3′ss. This treatment induced exon 9 skipping and reduced expression of the CHEK2 protein (Fig. 5e). SSOs targeting NSE or PE did not have any effect on *CHEK2* exon 9 inclusion (Fig. 5f). Skipping of exon 9, but not NSE, was dramatically increased in cells lacking SF3B1 (Fig. 5g). To address why exogenous expression of CHEK2 was elevated in cells lacking U2AF35 as compared to controls (Fig. S1e), we cotransfected HEK293 cells with the CHEK2-repressing SSO and a CHEK2-expressing plasmid (Fig. S1f,g). Reduced endogenous CHEK2 was associated with a significant increase of exogenous CHEK2 also in U2AF-proficient cells, pointing to a tight homeostatic regulation of the total CHEK2 protein in the cell.

Second, U2AF was required for full activation of *CDC25A* exon 6 (Fig. 5a), which encodes a serine residue (S178) phosphorylated by CHEK2/CHEK1. U2AF(35) was also required for inclusion of exon 3 of *CDC25B* and *CDC25C* (Fig. S3a,b), confirming microarray data^25^. *CDC25B* exon 3 encodes multiple phosphorylated residues, including S169; the isoform phosphorylated at S169 localizes to the centrosomes and accumulates during mitosis^26^. *CDC25C* exon 3 encodes T67 phosphorylated by cdc2/cyclin-B as a part of the autoamplification loop^27^. Phosphorylated T67 in CDC25C creates a binding site recognized by the WW domain of PIN1^27^, which sustained activation of another U2AF-repressed NMD switch exon (Fig. S4b), possibly modifying catalytic activity of this abundant peptidyl-prolyl isomerase. Finally, cyclin B1 and B2 mRNAs were upregulated in cells lacking U2AF35 as well as cyclin B1-interacting protein (CCNB1IP1), although their RNA processing patterns did not appear altered (Fig. 5a). Cyclin B upregulation was associated with a detained *CDK1* intron (Fig. S4c), which may be spliced post-transcriptionally^14^.

ATM recruitment to DSB is facilitated by the MRN complex, consisting of MRE11, RAD50 and NBN^22^. *NBN* showed no obvious RNA processing changes in cells lacking U2AF35, but *RAD50* mRNA was downregulated, possibly through activation of a NMD switch exon and/or additional splicing alterations (Fig. S5a-c and Fig. S2). The last *MRE11A* exon was upregulated, possibly as a result of a promotion of distal alternative polyadenylation site in depleted cells, which is used in most cell types, but not in B cells^28^. DEXSeq analysis did not detect significant RNA processing changes in transcripts encoding other members of the phosphatidylinositol 3 kinase-like family of serine/threonine kinases (*ATR* and *PRKDC*), nor in *BRCA1/2, RNF168* and the ATM interactor *ATMIN*. Additional ATM interacting partners with altered exon or gene expression included *RPS6, SRSF1* and other SR proteins, *EP300*, *RPA2, BLM, FANCD2* and *FANCI, PPP2R5C* and *PPP2R5D*, and *SMC3*, a central component of the cohesin complex (Fig. S2).

Depletion of U2AF35 was associated with preferential alterations of genes/exons involved in chromatin modification^11^, which have numerous functional links to ATM signalling (Fig. S2)^22^. For example, the INO80 chromatin remodelling complex is phosphorylated by ATM and is functionally linked to checkpoint regulators, including CHEK2^29^. U2AF inhibited INO80C isoforms with 54-nt exons^11^, which are likely to be involved in heterodimer formation with ACTR5. U2AF35 depletion altered expression of multiple INO80 components, including ACTR5, ACTL6A and RUVL2B^11^. Many INO80 subunits are also preferentially located in telomeres^30^. U2AF was required for full inclusion of *TERF1* exon 7 in the mRNA (Fig. S6a), thus controlling the relative abundance of TRF1 (exon 7+)/PIN2 (exon 7−) isoforms, important components of the telomeres-protecting shelterin complex. Exon 7 encodes multiple phosphorylated serine residues and both isoforms can heterodimerize through the dimerization domain^31^. TRF1 binding to telomeres is also promoted by ATM inhibition whereas ATM-mediated phosphorylation impairs TRF1 interaction with telomeric DNA^32^. TRF1 association with telomeres is also negatively regulated by RAD50^32^. TRF1-interacting TIF2, another shelterin protein localized in nuclear matrix, exists in at least two isoforms produced by alternative splicing, termed TIN2*S* and TIN2*L*^33^. TIN2*L* contains an extra NM binding domain and associates more strongly with the nuclear matrix than TIF2*S¸*which is encoded by a transcript with retained 3′ introns that form a long 3′ untranslated region^33^. This mRNA isoform was repressed by U2AF (Fig. S6b).

Collectively, these results show that the MRN/ATM-CHEK2-CDC25-cdc2/cyclin B axis is at the centre of the U2AF(35)-mediated control of DDR, although the U2AF regulation extends into additional ATM substrates, mainly involved in chromatin modification and telomere activity.

### U2AF preferentially controls RNA processing of transcripts involved in leukemia-associated fusions

CHEK2 phosphorylates PML (Promyelocytic Leukemia) and appears to require PML for subsequent autophosphorylation^34^. Depletion of U2AF35 promoted the use of proximal alternative polyadenylation site of *PML*, leading to the upregulation of the shortest PML isoform, which lacks the last exon coding for the nuclear export signal (Fig. S7a). The long and short PML isoforms have distinct functions; for example, nuclear PML isoforms, but not the cytoplasmic isoform, are positive regulators of IFNγ signalling^35^. The C-terminus of the longest isoform specifically interacts with AML1 to enhance AML1-mediated transcription^36^, suggesting that U2AF deficiency could impair PML-AML1 interactions. PML also binds PIN1 and this interaction promotes PML degradation in a phosphorylation-dependent manner^37^. U2AF depletion increased a *PIN1* NMD exon (Fig. S4b), potentially limiting expression of this abundant peptidyl-prolyl isomerise, which interacts with many phosphoproteins to regulate mitosis, including phosphorylated CDC25^38^. Apart from PML, U2AF35 depletion upregulated other RARA partners, including *NPM1* (Fig. S7b). This event was associated with promotion of a proximal polyadenylation site and increased the abundance of shorter transcripts. An alternatively spliced exon of *BCOR,* a BCL6 corepressor that forms BCOR-RARA fusions^39^ and interacts with several histone deacetylases to increase BCL6 transcriptional repression^40^, was also downregulated (Fig. S7c).

Interestingly, the overlap between U2AF35-sensitive genes/exons and 1,187 genes involved in cancer-associated gene fusions^41^ and 300 genes involved in recurrent chromosome translocations^42^ was greater than expected, with more significant P values for genes with differentially used exons than those implicated by the Cufflinks algorithm (Table 1). Similarly, sharing of genes frequently mutated in the myelodysplastic syndrome^43^ and genes differentially expressed upon U2AF35 depletion^11^ was significantly higher than expected (P < 0.01, hypergeometric test). Thus, RNA processing of transcripts involved in cancer-associated gene fusions and chromosome translocations is preferentially regulated by U2AF.

**Figure 6:**
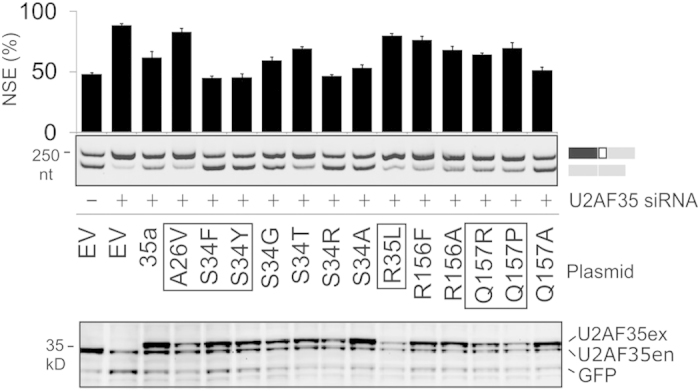
Rescue of NSE repression by cancer-associated mutations in U2AF35. Rescue of U2AF(35)-dependent NSE splicing of the C minigene by zinc finger 1 and 2 substitutions in U2AF35 (*upper panel*). All substitutions were made in the *U2AF1a* construct (35a)^11^. Cancer-associated mutations (*bottom*) are boxed; splice products are to the right. Error bars are SDs of 2 transfections. Immunoblot with U2AF35 and GFP antibodies is shown in the *lower panel*; ex, exogenous; en, endogenous U2AF35.

To test the function of cancer-associated *U2AF1* mutations in NSE splicing, we performed reconstitution experiments with wild-type and mutated U2AF35 constructs that were cotransfected with the C minigene into cells (mock)-depleted of U2AF35 (Fig. 6). NSE activation was repressed by either U2AF35 isoform to a similar extent, as well as U2AF35a containing cancer-associated substitutions of S34 in the zinc finger 1 domain, the most frequently mutated U2AF35 residue in cancer^7^. In contrast, only a partial rescue was achieved by substitutions of Q157 in the second zinc finger domain where these mutations are less frequent. Other S34 mutations failed to fully reconstitute the defect, including S34T and substitutions with small amino acids, although a large residue at this position (S34R) was efficient, suggesting that the size of this residue is critical for ligand binding. Thus, the identity of the residue at position 34 of U2AF35 was important for NSE recognition.

Finally, we detected a low degree of NSE activation in diverse human tissues, both in hexamer-primed samples and polyadenylated transcripts (Fig. S8a). The proportion of NSE-containing RNAs was on average higher in leukemic cells than in normal cells, with some samples exhibiting very high levels (Fig. S8b,c), potentially contributing to reduced ATM expression in cancer cells. We also examined NSE inclusion in polyadenylated RNAs extracted from a panel of lymphoblastoid cell lines exposed to cold and heat shock at the indicated temperatures prior to lysis (Fig. S8d,e). Interestingly, NSE was activated to a minor extent by exposing cells to 42 °C but not at subphysiological temperatures (Fig. S8d), suggesting that markedly higher NSE inclusion levels in malignant cells are unlikely to be explained by a cold shock encountered during storage of patients’ samples. Since proteomic profiling of Jurkat cells exposed to a heat stress at 43 °C revealed diminished expression of several proteins including U2AF35a^44^, these results further support U2AF35 as a specific NSE repressor.

## Discussion

Here we have identified an alternative splicing-coupled NMD switch exon critical for ATM expression (Figs 1 and 3) and examined its importance in cancer risk (Fig. 2, Fig. 6 and S8). We have also shown how inclusion of this exon in mature transcripts is influenced by intronic haplotypes and RNA-binding proteins involved in 3′ss selection (Figs 2 and 4h). Finally, we have identified SSOs that modulate activation of this exon by targeting its regulatory sequences and proposed a novel antisense strategy for modifying gene expression. These results significantly expand currently known links between RNA processing and DDR pathways (Fig. 5 and S2).

U2AF-repressed exons have a distinct 3′ss organization and response to U2AF-related proteins as compared to U2AF-activated exons^11^, suggesting that the NSE repression involves direct RNA binding. This is supported by the observed NSE activation on exogenous transcripts that do not undergo NMD and by the SSO-induced NSE blockage (Figs 2 and 4). NSE lacks AG dinucleotides between the predicted BPS and 3′ss, its AG exclusion zone is longer than the average and has an unusual stretch of 5 conserved guanines upstream of the BPS, which may contribute to stable secondary structures across 3′ss that might be required for the inhibition. The adenine-rich 3′ portions of both NSE and PE are more conserved in evolution than their 5’ parts (Fig. 4a), potentially providing important ligand interactions, given the propensity of adenine to occupy unpaired positions in structured RNAs. Although direct RNA binding appears to be the simplest explanation for exon repression by U2AF, U2AF35 depletion led to downregulation of several proteins involved in NMD (Table S4), which may contribute to NSE activation on endogenous transcripts. Physical interactions between U2AF65 and the C-terminus of TRF1^45^ or other components of the ATM signalling network^46^ could also participate in NSE regulation. A challenge ahead will be to gain insight into mechanisms of NSE repression in normal and cancer cells where the NSE control appears to be compromised, potentially altering ATM expression and DDR in the haplotype-dependent manner (Fig. 2 and S8b,c).

Apart from *U2AF1/U2AF2*, additional genes involved in 3′ss selection have been found mutated in cancer^7^. Interestingly, chronic lymphocytic leukemias with *SF3B1* mutation were associated with a cryptic 3′ss activation of *ATM* exon 46, leading to ATM truncation^47^. Splicing of an *EZH2* exon as a result of cancer-associated *SRSF2* mutation was implicated in impaired hematopoietic differentiation^48^ and the same NMD exon was upregulated also in cells lacking U2AF35 (Fig. S5d). Whether these exons are targets of a common 3′ss recognition pathway underlying leukemogenesis remains to be established.

Because NSE activation may restrict ATM expression both in normal and cancer cells (Figs 1,2, and S8) and ATM is a limiting factor in the DDR pathway^22^, cytosine at rs609261 may confer a relative ATM deficiency also in the germline. ATM kinase activity appears to be a good predictor of A-T severity, however, the diversity of A-T alleles does not fully account for the spectrum of clinical symptoms, arguing for the role of additional factors^49^. Our results suggest that genes involved in NSE activation and natural variants modulating NSE inclusion (Figs 1, 2c–f and 4h) could modify the phenotypic complexity of A-T and also A-T heterozygotes who lack overt clinical features but may display increased radiosensitivity and cancer risk^50^, ie. phenotypes influenced by the critical U2AF-regulated pathway in the ATM signalling network (Fig. S2). Although the SSO-induced repression of endogeneous NSE transcripts was observed also in untreated cells (Fig. 3b) and NMD transcripts with the relative abundance as low as ~1% can dramatically contribute to the mRNA consumption^51^, it remains to be tested if their reduction can lead to a sustained increase of ATM protein levels. This approach may have a potential to alleviate phenotypic consequences of leaky A-T alleles in a mutation-independent manner, particularly in patients carrying the C allele at rs609621.

Our results also show that NSE activation is on average more efficient in Caucasians than in Asian populations as a result of the higher frequency of the C allele at rs609621 in the former (Fig. 2). This differential NSE control may potentially contribute to persistently lower mortality rates for common malignancies in Asian populations than in Caucasians^52^,^53^. rs609261 is only ~35 kb downstream of rs2235006, which is located in a region of minimal meiotic recombination^54^ and was associated with a high risk (OR 11.2) of chronic lymphocytic leukemia^55^. This study genotyped 1467 coding nonsynonymous SNPs in 865 candidate genes and implicated variants in genes encoding the ATM-BRCA2-CHEK2 DDR axis as the most significant risk pathway. Asian cancer patients were also found to exhibit a lower prevalence of some gene fusions than Caucasians^56^, potentially reflecting their capacity to respond to DSBs.

Our study also highlights current limitations of incomplete transcript annotation and the importance of examining cryptic exons in RNA-Seq data. Although RNA-Seq is a powerful tool to examine global transcriptome, rigorous standards that correctly evaluate biological and statistical significance of the observed alterations in RNA processing are yet to be implemented. Given a high stringency of the default DEXSeq algorithm^11^,^57^, we cannot exclude the existence of additional biologically important RNA processing events regulated by U2AF. For example, promotion of a proximal polyadenylation site in *CHEK1* in depleted cells coupled with upregulation of 24-nt and 27-nt exons in *CLASP1*, events observed in genomic browsers, would implicate the ATM apoptotic pathway. This pathway is of particular interest in the myelodysplastic syndrome where myeloid progenitors show susceptibility to the programme cell death, mutations in 3′ss recognition genes are particularly frequent^7^, and deregulation of genes involved in ATM signalling is associated with more advanced clinical stages^58^. Interestingly, *U2AF1* mutations were found to be more frequent in later stages and were associated with shorter survival^59^.

Finally, our work demonstrates efficient repression of a critical NMD switch exon in *ATM* by SSOs that target regulatory motifs of competing pseudoexons and are capable of increasing ATM protein levels (Figs 3a–d, 4 and S1). The proposed strategy may be combined with genome editing such as CRISPR-Cas9 to alter splicing regulatory motifs or protein binding sites in introns and should also help us to find efficient intronic SSOs with desired outcomes for RNA processing. The search for such SSOs is more challenging than for those targeting human exons. For example, most SSOs systematically covering *SMN2* exon 7 stimulated exon skipping, which is required for antisense therapy of spinal muscular atrophy, however, ~20% increased exon inclusion^60^. Conversely, stimulation of intron splicing was found only for ~10% of SSOs targeting *INS* intron 1 while the majority failed to show this effect^61^. The versatility of intronic or exonic SSOs to modulate splicing in either direction exploits a much higher information content of auxiliary splicing sequences in humans as compared to lower organisms. Future SSO design strategies should be therefore greatly facilitated by global pre-mRNA folding and RNA crosslinking and immunoprecipitation studies. For example, unlike the SSO that efficiently blocked the NSE 3′ss (SSO-NSE3, Fig. 3a,b), a partially overlapping morpholino extending only 7-nt into NSE failed to repress the same 3′ss to rescue splicing of mutation IVS28-159A>G^62^, despite targeting U2AF binding sites (Fig. 4a). This suggests that the morpholino blocked access to structures or motifs that are not responsible for exon activation, but exon repression, in agreement with our finding (Fig. 1a–c). Thus, administration of antisense-based RNA processing activators or inhibitors that target or avoid binding sites of splicing factors in introns could be exploited therapeutically to shape beneficial or detrimental consequences of NMD in cancer cells.

## Methods

### Plasmid constructs

*ATM* minigenes were prepared by cloning ~0.9-kb amplicons into *Xho*I/*Xba*I sites of the *U2AF1* construct described earlier^11^. Cloning primers are shown in Table S1. Full inserts were sequenced to confirm the identity of intended changes and exclude undesired mutations. The PUF60 expression vector was described previously^11^. The hnRNP A1 construct was a generous gift of Gideon Dreyfuss (University of Pennsylvania).

### Cell cultures and transfections

Cell cultures were maintained in standard conditions in DMEM supplemented with 10% (v/v) bovine calf serum (LifeTechnologies). Depletion of U2AF subunits and U2AF35 isoforms with small interfering RNAs (siRNAs) and splice-switching oligonucleotides (SSOs), were carried out following a time course experiment that established depletion levels of each isoform, as described^11^. All oligo(ribo)nucleotides are listed in Table S1. Transfections were carried out in 6- or 12-well plates using jetPRIME (Polyplus) according to manufacturer’s recommendations, as described in detail^11^. The cells were harvested 48 hrs after the second hit, except for those exposed to IR, which received a single hit. For SF3B1 depletion, HEK293 cells were exposed to a siRNAs mixture (S23850, S23852, S223598, LifeTechnologies) and were harvested 48 hrs later.

### RNA-Seq

Analysis of differential exon usage was performed using DEXSeq (v. 1.12.1)^57^, based on q-values less than 0.05, as described in detail^11^. Differential gene and isoform expression between replicated sample sets was analyzed with Cufflinks (v. 2.1.1)^63^, which normalizes the reads using a fragments per kilobase of exon model per million reads measure. Selection of significantly differentially expressed genes was made on the basis of FDR-adjusted P-values (q < 0.05). Cells, RNA extraction and library preparation was as described previously^11^.

### NSE expression in human tissues and cell lines

The FirstChoice human total RNA survey panel containing total RNA samples from 19 different tissues was purchased from LifeTechnologies. Each tissue sample contained a pool of RNAs from different donors. Lymphoblastoid cell lines exposed to cold and heat shock were described previously^64^. Total RNA samples were reverse transcribed with the Moloney murine leukemia virus reverse transcriptase (Promega) and random hexamer or oligo-d(T) primers. cDNA samples were amplified using primers shown in Table S1. Total RNA extracted from leukocytes from bone marrow samples of randomly selected patients with acute myeloid leukemia or chronic myelomonocytic leukemia was reverse transcribed with random hexamer primers. The study was approved by the National Research Ethics Service (UK) Committee South West.

### Splice-switching oligonucleotides

SSOs were designed to maximize interactions with single-stranded regions^21^ and avoid secondary structures predicted by Mfold^65^. All SSOs were purchased from Eurofins, diluted in water and their aliquots were stored at −80 °C. Their sequences are in Table S1. All transfections were carried out with jetPRIME (Polyplus) according to manufacturer’s recommendations.

### Exposure of cell cultures to ionizing irradiation (IR)

(Mock)-depleted HEK293 cells were exposed to IR 48 hours after the first hit using a Gulmay Medical (X-Strahl) D3225 Orthovoltage X-ray system at a dose-rate of 0.63 Gy min^−1^ at room temperature. The actual dose rate was monitored by a constancy meter. Cells were harvested as indicated in figure legends.

### Immunoblotting

Preparation of cell lysates and immunoblotting was carried out as described^11^. Antibodies against ATM (D2E2), ATM-pS1981 (D6H9), CHEK2 (D9C6) and CHEK2pThr68 (C13C1) were purchased from the Cell Signaling Technology, Inc. RBM39 (CAPERa) antibodies were purchased from Thermo Fisher Scientific (PA5-31103). Antibodies detecting X-press tag, U2AF35, U2AF65, and tubulin were as described previously^11^. SF3B1 and FUBP1 immunoblotting was performed with anti-SAP155 (D138-3, MBL) and anti-FBP (GTX104579, GeneTex) antibodies, respectively. ATM signal intensity was measured by densitometry using ChemiDoc XRS (BioRad) and serial dilutions of pooled lysates on SDS-PAGE to determine the linear dynamic range of protein loading. Densitometric signal was normalized to total blot-transferred protein (Fig. S1a,b) since U2AF35 depletion alters expression of hundreds of exons, many in housekeeping genes^11^.

## Additional Information

**How to cite this article**: Kralovicova, J. *et al.* Exon-centric regulation of ATM expression is population-dependent and amenable to antisense modification by pseudoexon targeting. *Sci. Rep.* **6**, 18741; doi: 10.1038/srep18741 (2016).

## Supplementary Material

Supplementary Information

## Figures and Tables

**Table 1:** U2AF35-dependent transcripts are more common than expected among genes involved in cancer-associated gene fusions and recurrent chromosomal translocations.

Database	Reference	Number of genes^1^	Overlap with U2AF(35)-sensitive exons^2^	P-value /representation factor^3^	Overlap with U2AF(35)-sensitive transcripts^2^	P-value /representation factor^3^
ChimerDB 2.0	41	1,187	66	P < 0.00004/1.7	204	P < 0.02/1.1
Genes involved in recurrent structural abnormalities in cancer	42	300	19	P < 0.006/1.9	56	P < 0.05/1.2

^1^Gene lists were downloaded on 2 April 2014.

^2^Exon- and gene-level analysis of RNA-Seq data was carried out for 23,263 genes using DEXSeq and Cufflinks, respectively, as described in detail^11^.

^3^Number of overlapping genes divided by the expected number of overlapping genes drawn from two independent groups. A representation factor >1 indicates a greater overlap than expected of two independent groups, the value <1 indicates less overlap than expected. P-values were derived by hypergeometric tests.
