# Corrigendum: Exon-centric regulation of ATM expression is population-dependent and amenable to antisense modification by pseudoexon targeting

**DOI:** 10.1038/srep25256

**Published:** 2016-05-09

**Authors:** Jana Kralovicova, Marcin Knut, Nicholas C. P. Cross, Igor Vorechovsky

Scientific Reports 6: Article number: 18741;10.1038/srep18741 Published online: 01062016; Updated: 05092016

In the original version of this Article, some instances of ‘rs609261’ were incorrectly written as “rs609621”. These errors have now been corrected in the PDF and HTML versions of the Article.

