# Supplementary material for: Exon-centric regulation of ATM expression is population-dependent and amenable to antisense modification by pseudoexon targeting
Source: Sci Rep. 2016 Jan 6;6:18741. doi: 10.1038/srep18741 (PMC4702124; doi:10.1038/srep18741)
Supplement: Supplementary Information [file srep18741-s1.pdf]

SUPPLEMENTARY INFORMATION

Exon-centric regulation of ATM expression is population-dependent and amenable to antisense modification by pseudoexon targeting

Jana Kralovicova[1], Marcin Knut[1], Nicholas C. P. Cross[1,2], Igor Vorechovsky[1]

[1]University of Southampton
Faculty of Medicine
Southampton SO16 6YD
United Kingdom

[2]Wessex Regional Genetics Laboratory
Salisbury Hospital
Salisbury SP2 8BJ
United Kingdom

SUPPLEMENTARY TABLES

Table S1 Synthetic DNAs/RNAs

RT-PCR primers	Sequence (5'-3')	Reference
ATM-F	GAGGGTACCAGAGACAGTGGGATGGC	This study
ATM-R	GGCTCATGTAACGTCATCAAT	This study
PL3	GGGAGACCCAAGCTGGCTA	[1]
CHEK2-E9-F	AGACCCAGCTCTCAATGTTG	This study
CHEK2-E9-R	TAGCTTCTTTCAGGCGTTTA	This study
CHEK2-E11F1	AGTGGTGGGGAATAAACG	This study
CHEK2-E11R	CAGCAGTCCACAGCACGGT	This study
CDC25B-F	CTCAGTCCAGCAGGCGTGTG	This study
CDC25B-R	GGTCTCTGGGCAAAGGCTTC	This study
CDC25A-F	CAGAAGCTGTTGGGATGTAG	This study
CDC25A-R	TCTCCATCGAGAAGGTCCAC	This study
CDC25C-F	TGGCTCAGGACCCAGTTTTA	[2]
CDC25C-R	TCTTCTGCCTGGTCTTCTCC	[2]
TIN2-F1	CCTGGCTTGGTTCGCTACC	This study
TIN2-R3	TGCTTCACCTGCTGGTAAAA	This study
TIN2-F6	GAAGAACATGCGATATACACA	This study
TIN2-R8	GTCTAAAACCAAGTCCCCTAT	This study
PIN1-F	GAGGGAAGATGGCGGACGAG	This study
PIN1-R	TCCTCCTTGGTCCGGGTGAT	This study
TERF1-F	GCAGCGGCAAAAGTAGTAGA	This study
TERF1-R	GTCTTGTTGCTGGGTTCCA	This study
RAD50-cr5-F	AAAATCATCAAAACAGCGAG	This study
RAD50-cr5-R	TGGAGCAAGTCGCAGTTTAG	This study
RAD50-E-F	GGCGACAGAAAGGTTATGAA	This study
RAD50-E-R	CGCCACAGGTCACGTATAAT	This study
**Cloning primers**		
*ATM-Xho*I	ATAGAATTCTCGAGGGGAGGGTTTTATTCTACTA	This study
*ATM-Xba*I	ATAGGGCCCTCTAGACTGTGGGGAGACTATGGTAA	This study
*CHEK2-EcoR*I	ATTAGAATTCTCTCGGGAGTCGGATGTTG	This study
*CHEK2-Not*I	ATTAGCGGCCGCGGTACATTTCTTTCGTGTTCA	This study
**Modified antisense oligonucleotides**		
SSO-NSE3	CUUCUAUGCAGCCAACCUGUAGACU	This study
SSO-NSE5	ACCUUUUUCUUCUAUGCAGCCAAC	This study
SSO-PE3	AUUUCCAAAAGUAUUCGAUGACUG	This study
SSO-PE5	UAUAUUACCUUAUUUCCAAAAGUA	This study
SSO-PEBP	CUGUAAAAGAAAAUAGAUGACUCAA	This study
SSO-PEdel	CUGUAAAAGAAAAUAGA	This study
SSO-CHEK2-ex9	ACUUACAAUUCCAAAACAAUAUAAU	This study
SSO-C	AGGUGCUCGCGGGUGG	[3]
SSO-MIR loop	AGUUGCUUCAUCU	[4]
**siRNA**		
U2AF35ab	GGCUGUGAUUGACUUGAAU	[5]
U2AF65	GCAAGUACGGGCUUGUCAA	[6]
RBM39	GGAUCUACUGUCAUUUGUA	[7]
PUF60	GCAGAUGAACUCGGUGAUG	[6]
UPF1	AAGAUGCAGUUCCGCUCCAUU	[8]

Table S2 Sequences of splicing reporter constructs mutated in NSE and PE

Mutation	Nucleotide sequence[1] (5′-3′)
WT	cagTCTACAGGTTGGCTGCATAGAAGAAAAAGgtagagttatttataat--------------------cttgtaaatcttggactttgagtcatctattttcttttacagTCATCGAATACT------------------------TTTGGAA
NSE 3′ssAG>GG	cggTCTACAGGTTGGCTGCATAGAAGAAAAAGgtagagttatttataat--------------------cttgtaaatcttggactttgagtcatctattttcttttacagTCATCGAATACT------------------------TTTGGAA
NSE 3′ssAG>GG/ins20	cggTCTACAGGTTGGCTGCATAGAAGAAAAAGgtagagttatttataattcttgacgttcacagatatacttgtaaatcttggactttgagtcatctattttcttttacagTCATCGAATACT------------------------TTTGGAA
PE-MIR	cagTCTACAGGTTGGCTGCATAGAAGAAAAAGgtagagttatttataat--------------------cttgtaaatcttggactttgagtcatctattttcttttacagTCATCGAATACTGGGCACAGAUGAAGCAACUUGCCCTTTGGAA
PE-MIR/ins20	cagTCTACAGGTTGGCTGCATAGAAGAAAAAGgtagagttatttataattcttgacgttcacagatatacttgtaaatcttggactttgagtcatctattttcttttacagTCATCGAATACTGGGCACAGAUGAAGCAACUUGCCCTTTGGAA

Legend: [1]NSE and PE are highlighted in gray, mutations/insertions are in red and underlined.

Table S3 Auxiliary splicing elements in NSE and PE

Pseudoexon	Allele	FAS-ESS[1]	PESS[1]	PESE[1]	RESCUE-ESE[1]	SF2/ASF ESE[1]	EIE density[1]
**NSE**	**C**	3.45	3.45	5	6	9.3	975.4
**NSE**	**T**	3.45	3.45	5	6	9.3	975.4
**PE**	**G**	4.17	0	0	3	0	403.8
**PE**	**A**	4.17	0	0	5	0	334.2
**Reference**	9	10	11	11	12	13	14

Legend: [1]Densities of the indicated splicing enhancers and silencers predicted in NSE and PE were computed as described[15]. Support vector machine scores for the best predicted BPS of NSE and PE[16] were 1.3 and 0.5.

Table S4 Summary of U2AF-regulated transcripts involved in NMD

**Transcript**	**Levels in depleted cells**
UPF3A	Down (q=0.002)
UPF3B[1]	Down (q=0.0006), skipping of a 39-nt exon
SMG7	Up (q=0.002)
SMG8	Up (q=0.01)
DHX34	Up (q=0.02)
CASC3	Up (q=0.04)
RNPS1	Up (q=0.03) through putative intronic APA
RBFOX2	Up (q=0.007)

[1] Differential exon inclusion in *UPF3B* was reported in patients with myeloid neoplasms[17].

SUPPLEMENTARY FIGURES

Figure S1 SSO-mediated NSE repression enhances ATM expression

Legend: **(A)** ATM expression induced by SSO NSE3 in HEK293 cells (homozygous for cytosine at rs609261). Cells were transfected with 30 nM of siRNA against U2AF35 on day 1 and with a mixture of the same siRNA (15 nM) and control and NSE3 SSOs (20 nM) on day 3 and 4. Cells were harvested 24 hrs later. Cell lysates were loaded on to SDS-PAGE as indicated. Ponceau S-stained nitrocellulose membranes are in *the upper panel*, immunoblots with antibodies shown to the right are in *the lower panel*. FUBP1 antibody was initially used as one of the loading controls, illustrating upregulation of a multifunction ubiquitously expressed protein in depleted cells. The upregulation was associated with skipping of an alternatively spliced *FUBP1* exon with a GC 5'ss consensus. This exon encodes at least two phosphorylated serine residues and is surrounded by highly conserved intronic sequences (panel D). **(B)** ATM expression in HeLa cells (homozygous for thymine at rs609261). Concentration of siRNAs against U2AF35 and SSOs was 60 nM and 50 nM, respectively. **(C)** NSE inclusion levels (%) in HEK293 and HeLa cells from experiment shown in panel A and B. **(D)** Genome browser view of *FUBP1* transcripts in cells depleted of U2AF35 (ab-) and controls (ctr) showing differential usage of exon 3. PhyloP, 100 vertebrates basewise conservation by PhyloP. **(E)** Expression of total and activated ATM upon SSO NSE3 treatment. HEK293 cells were (mock)-depleted of U2AF35, cotransfected with Xpress-tagged CHEK2 plasmids (30, 90 and 270 ng/mL) and NSE3/control SSOs, exposed to ionizing radiation (IR) and harvested 30 minutes later. Cell lysates were immunoblotted with the indicated antibodies. Final concentration of siRNA and SSOs was 30 nM. DNA from the empty vector was added to a final concentration of 270 ng/mL. Ex/enCHEK2, signal from exogenous and endogenous CHEK2 detected by antibody D9C6. **(F,G)** Increased expression of exogenous CHEK2 by an SSO targeting a NMD switch exon 9 (SSO CHEK2). Constant amounts of SSO CHEK2 were cotransfected with increasing amounts of Xpress-CHEK2 and constant amounts of GFP plasmids as transfection and loading controls **(F)** and *vice versa* **(G)**. Antibodies are to the right.

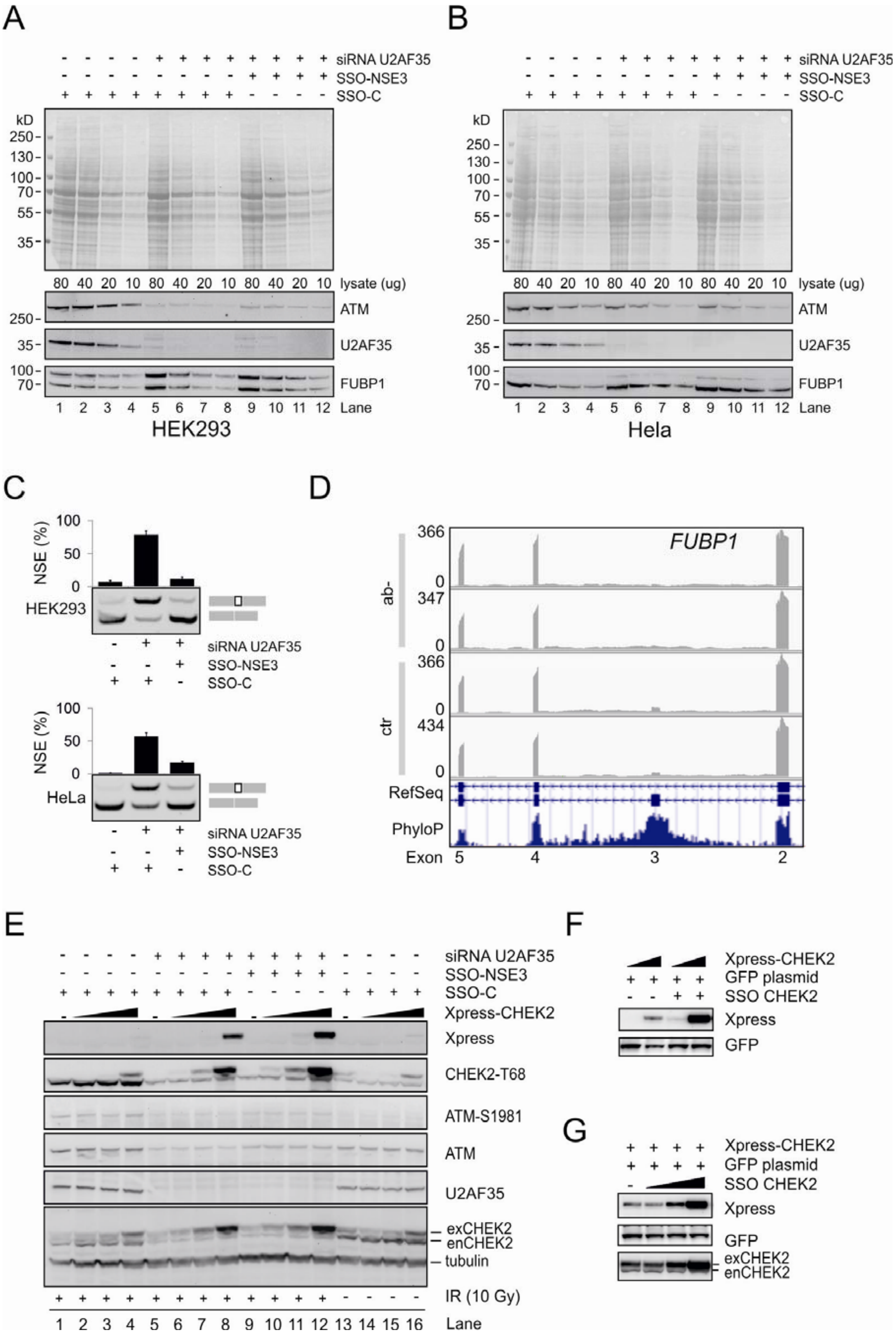
A
B
siRNA U2AF35
SSO-NSE3
SSO-C
kD
250
130
100
70
55
35
lysate (ug)
80 40 20 10 80 40 20 10 80 40 20 10
ATM
U2AF35
FUBP1
1 2 3 4 5 6 7 8 9 10 11 12 Lane
HEK293
Hela
C
D
NSE (%)
100
50
0
HEK293
HeLa
FUBP1
366
347
434
ab-
ctr
RefSeq
PhyloP
Exon 5 4 3 2
E
F
G
Xpress-CHEK2
Xpress
CHEK2-T68
ATM-S1981
ATM
U2AF35
exCHEK2
enCHEK2
tubulin
IR (10 Gy)
1 2 3 4 5 6 7 8 9 10 11 12 13 14 15 16 Lane
GFP plasmid
SSO CHEK2
GFP

Figure S2 Map of U2AF-regulated functional ATM interactions

Legend: U2AF-regulated ATM signalling network is highlighted by red arrows/pink background. Genes up-/down-regulated in cells depleted of U2AF35 are shown in red/green, respectively. Genes exhibiting significantly altered exon usage are shown in yellow. The ATM signalling map shows ATM-interacting proteins (white)/protein complexes (gray) (adapted from ref. [18]). Arrows correspond to activation, T-shaped edges to inhibition and circles denote unknown regulations. Containment links are shown as green edges. Domain structure of ATM is shown in the middle. Proteins upregulated in cells lacking U2AF(35) and related to SR and MCM components of the network are shown at the bottom (asterisks).

Fig. S2

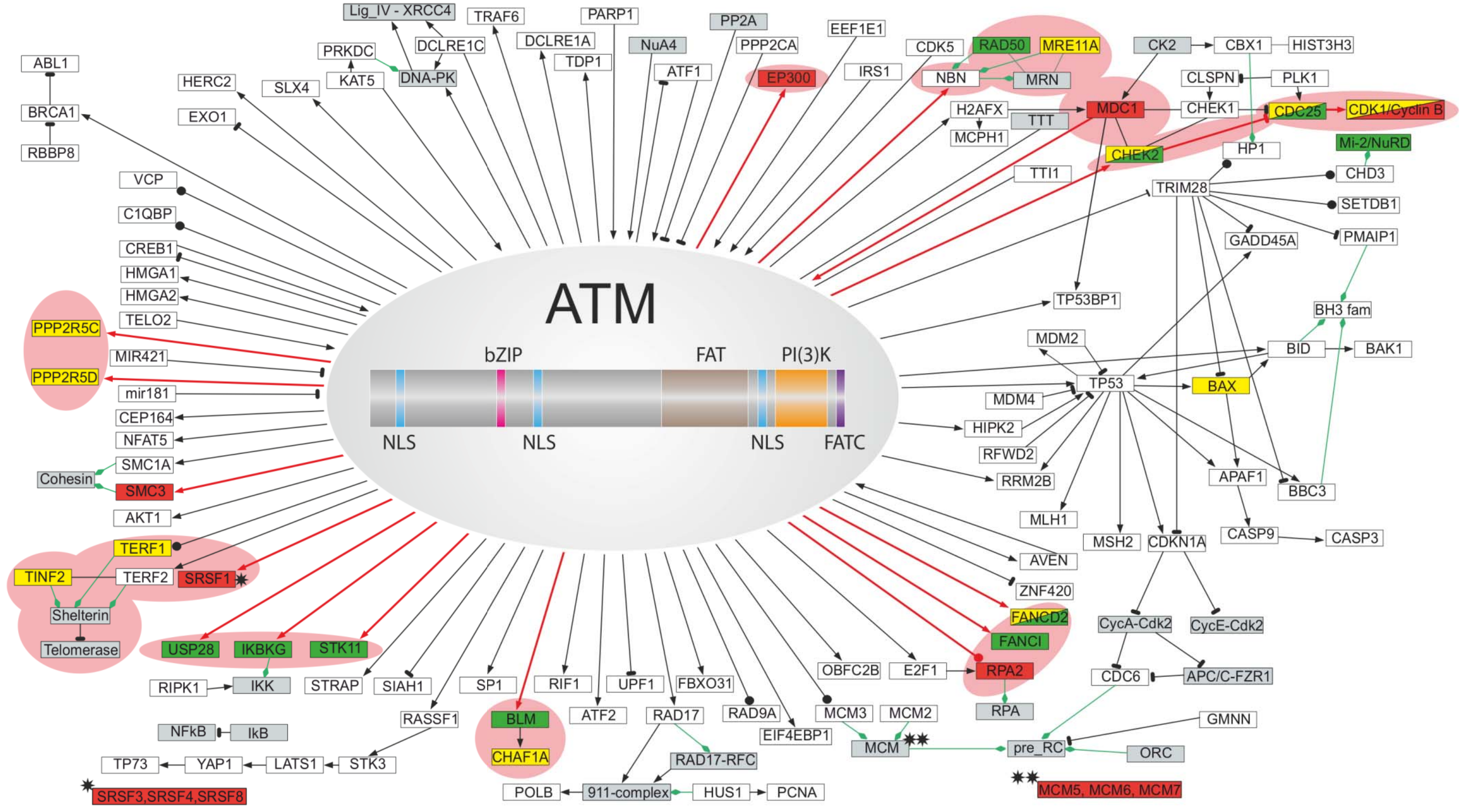
ATM
bZIP
FAT
PI(3)K
NLS
NLS
NLS
FATC
ABL1
BRCA1
RBBP8
HERC2
EXO1
SLX4
KAT5
PRKDC
DNA-PK
Lig_IV - XRCC4
DCLRE1C
TRAF6
DCLRE1A
TDP1
PARP1
NuA4
ATF1
PP2A
PPP2CA
EP300
EEF1E1
IRS1
CDK5
NBN
RAD50
MRE11A
MRN
H2AFX
MCPH1
TTT
TTI1
MDC1
CK2
CBX1
HIST3H3
CLSPN
PLK1
CHEK1
CDC25
CDK1/Cyclin B
CHEK2
HP1
Mi-2/NuRD
CHD3
TRIM28
SETDB1
GADD45A
PMAIP1
TP53BP1
BH3 fam
BID
BAK1
MDM2
TP53
BAX
MDM4
HIPK2
RFWD2
RRM2B
MLH1
MSH2
CDKN1A
APAF1
BBC3
CASP9
CASP3
AVEN
ZNF420
FANCD2
FANCI
RPA2
RPA
CycA-Cdk2
CycE-Cdk2
CDC6
APC/C-FZR1
GMNN
pre_RC
ORC
MCM5, MCM6, MCM7
OBFC2B
E2F1
MCM3
MCM2
MCM
EIF4EBP1
RAD9A
FBXO31
RAD17
RAD17-RFC
911-complex
HUS1
PCNA
POLB
UPF1
ATF2
RIF1
SP1
BLM
CHAF1A
SIAH1
RASSF1
STRAP
STK3
LATS1
YAP1
TP73
SRSF3,SRSF4,SRSF8
NFkB
IkB
RIPK1
IKK
USP28
IKBKG
STK11
TERF1
TERF2
TINF2
SRSF1
Shelterin
Telomerase
AKT1
SMC3
SMC1A
Cohesin
NFAT5
CEP164
mir181
MIR421
PPP2R5C
PPP2R5D
TELO2
HMGA2
HMGA1
CREB1
C1QBP
VCP

Figure S3 Exon usage in *CDC25B* and *CDC25C* in cells depleted of U2AF35

Legend: Genomic browser views of RNA-Seq data in control (ctr) and depleted (ab-) cells (*left panels*). PCR primers are shown by arrows, differentially used exons are denoted by black rectangles. RefSeq exon annotation is shown at the bottom. Validation of RNA-Seq data using RT-PCR with RNA extracted from cells depleted of each U2AF subunit and U2AF-related proteins is shown in the *right panels*. RNA products are schematically shown to the right.

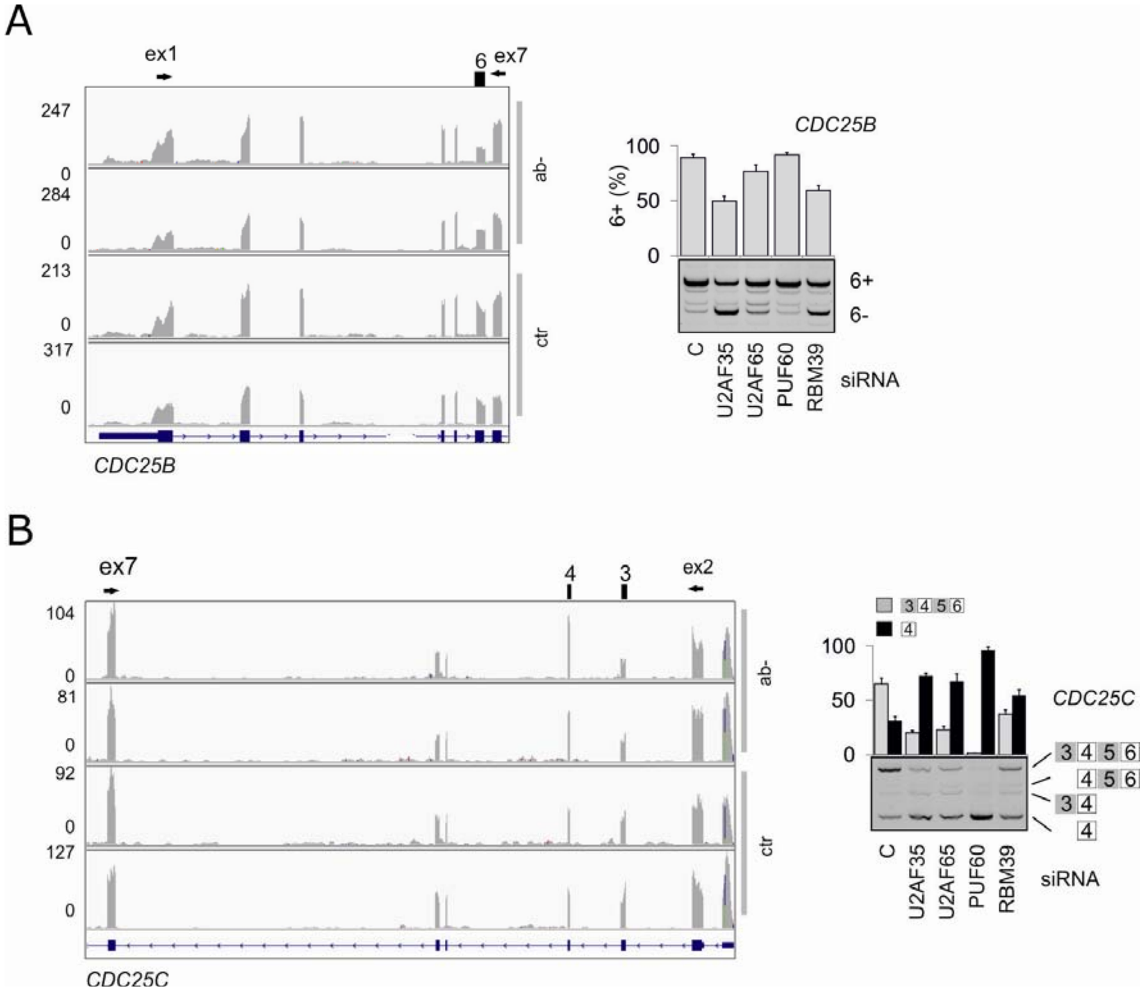

Figure S4 U2AF-regulated exon usage in *TTK, PIN1*and *CDK1*

For legends to Figs. S4-S7, see Fig. S3.

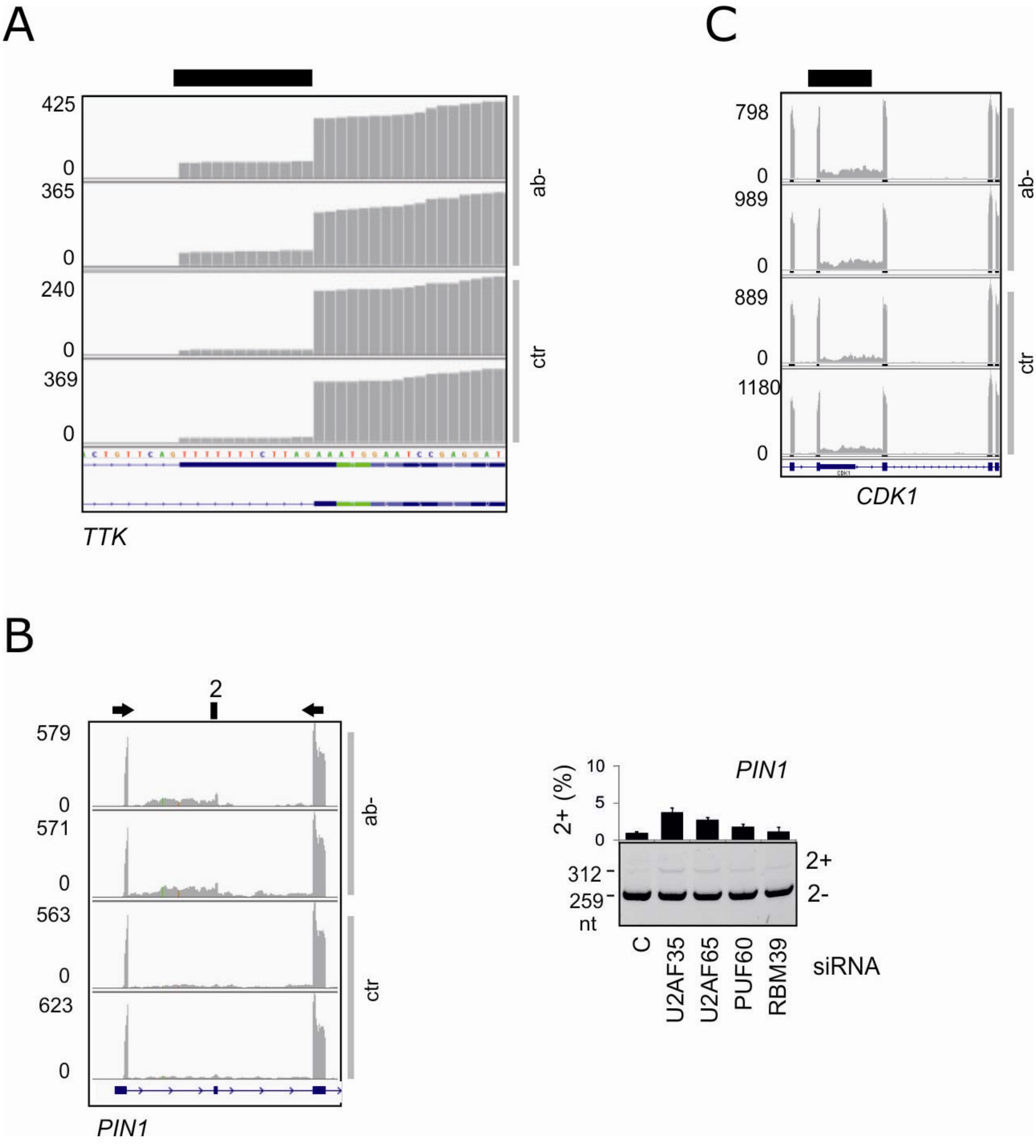

Figure S5 RNA processing of *RAD50* and *EZH2* in depleted cells

Legend: The last track in panel C shows proximal and distal alternative polyadenylation sites determined by 3' end sequencing[19].

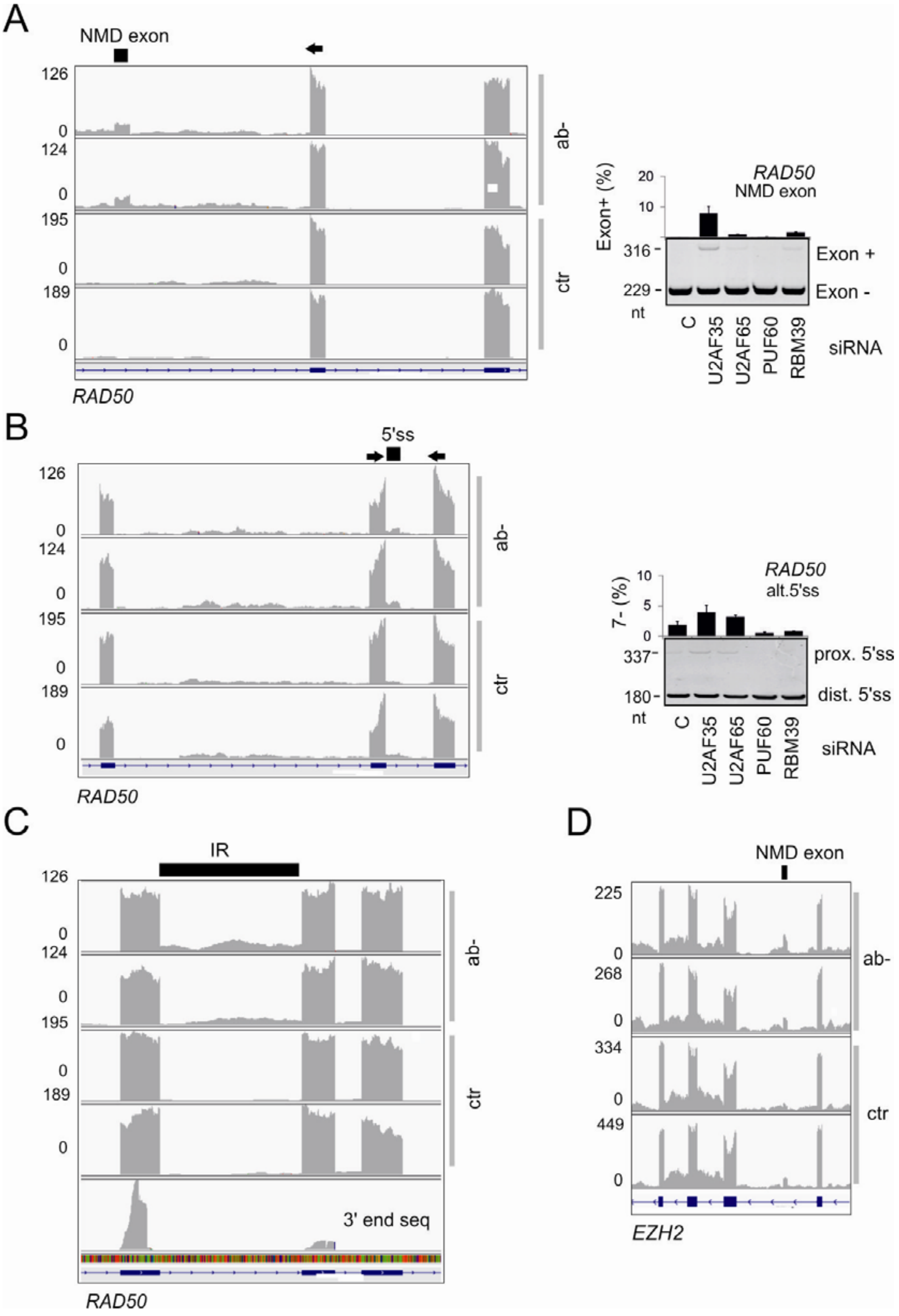

Figure S6 U2AF(35)-controlled exon usage of the peptidyl-prolyl isomerise PIN1 and components of the shelterin complex

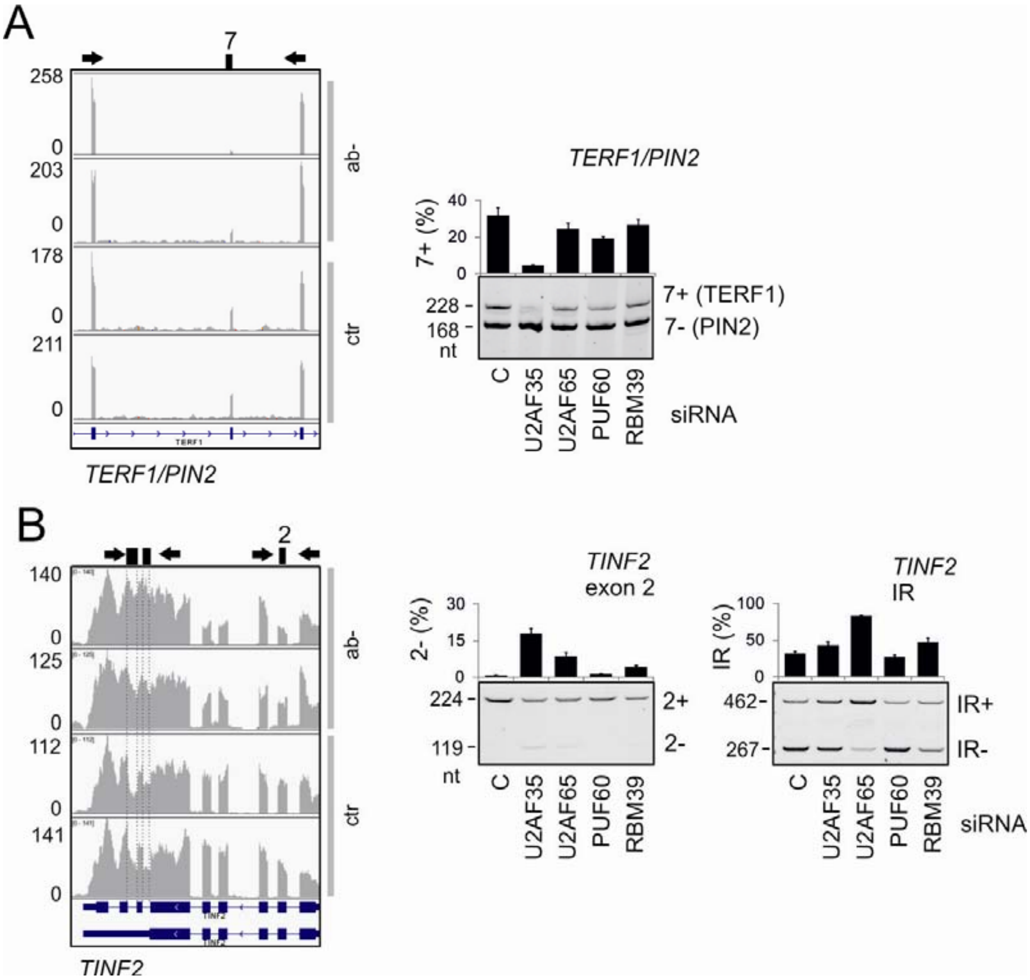

Figure S7   U2AF control of RARA fusion partners
Legend: APAprox, APAdist – proximal and distal alternative polyadenylation sites were determined by 3' end sequencing [19].

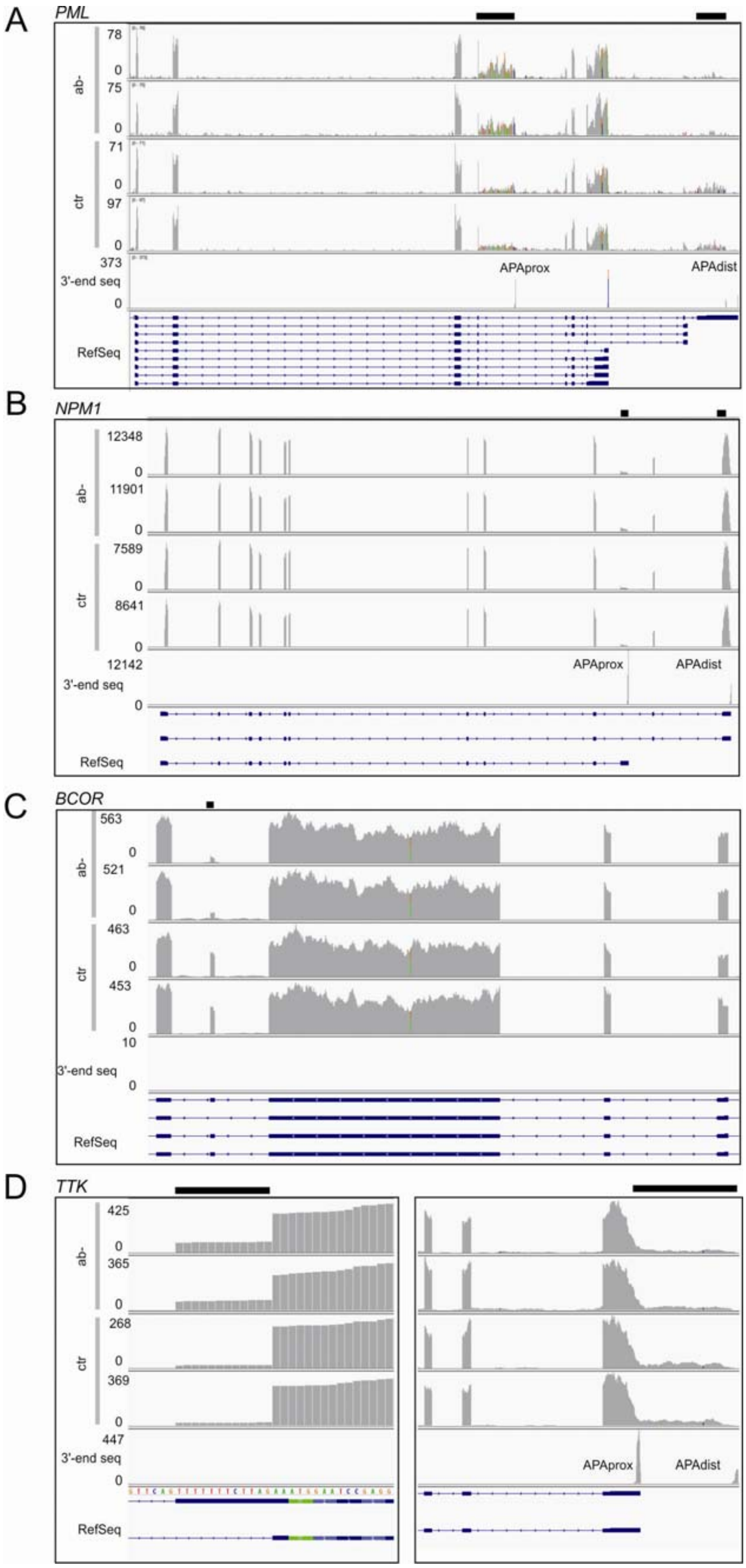

Figure S8 NSE activation in normal tissue and leukaemic cells

Legend: NSE inclusion levels were measured in 19 human tissues (**A**) and 17 AML/CMML bone marrow samples (**B**) using primers ATM-F and ATM-R (Table S1; shown as arrows in Fig. 1a). Exon inclusion was quantified as described[20]. (**C**) Means in normal tissues and leukaemia cells (horizontal bars in box plots) were compared with an unpaired t-test. (**D,E**) Inclusion levels of three U2AF-repressed (**D**) and three -activated (**E**) exons in two lymphoblastoid cell lines (top). Cells were exposed to cold and heat shock at the indicated temperatures as described[20]. ES, exon skipping; EI, exon inclusion. Transcript symbols are shown below each panel.

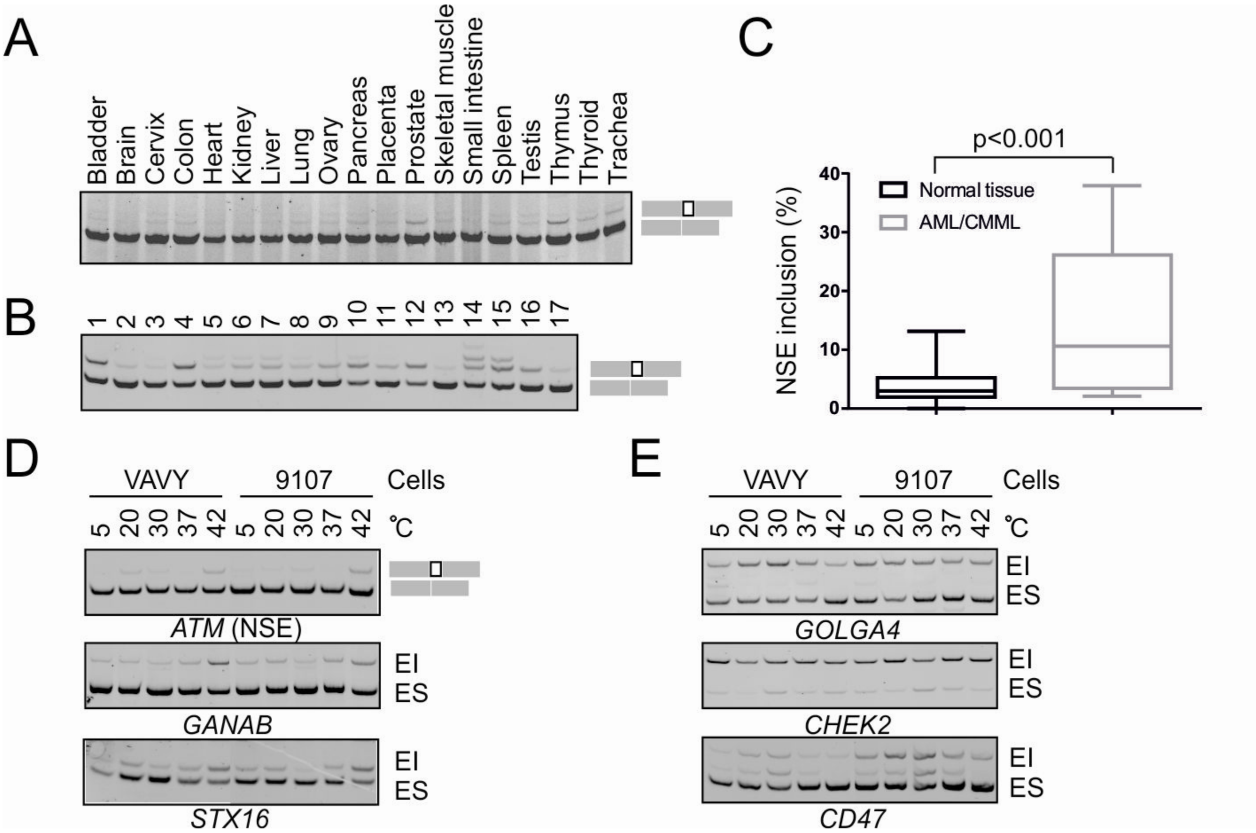

REFERENCES TO SUPPLEMENTARY INFORMATION

1. Kralovicova, J., Haixin, L. & Vorechovsky, I. Phenotypic consequences of branchpoint substitutions. *Hum. Mutat.* **27**, 803-813 (2006).
2. Pacheco, T.R., Moita, L.F., Gomes, A.Q., Hacohen, N. & Carmo-Fonseca, M. RNA interference knockdown of hU2AF35 impairs cell cycle progression and modulates alternative splicing of Cdc25 transcripts. *Mol. Biol. Cell* **17**, 4187-4199 (2006).
3. Kralovicova, J., *et al.* Optimal antisense target reducing INS intron 1 retention is adjacent to a parallel G quadruplex. *Nucleic Acids Res.* **42**, 8161–8173 (2014).
4. Kralovicova, J., Patel, A., Searle, M. & Vorechovsky, I. The role of short RNA loops in recognition of a single-hairpin exon derived from a mammalian-wide interspersed repeat. *RNA Biol* **12**, 54-69 (2015).
5. Pacheco, T.R., *et al.* Diversity of vertebrate splicing factor U2AF35: identification of alternatively spliced U2AF1 mRNAS. *J. Biol. Chem.* **279**, 27039-27049 (2004).
6. Hastings, M.L., Allemand, E., Duelli, D.M., Myers, M.P. & Krainer, A.R. Control of pre-mRNA splicing by the general splicing factors PUF60 and U2AF. *PLoS ONE* **2**, e538 (2007).
7. Kralovicova, J. & Vorechovsky, I. Allele-dependent recognition of the 3' splice site of *INS* intron 1. *Hum. Genet.* **128**, 383-400 (2010).
8. Mendell, J.T., ap Rhys, C.M. & Dietz, H.C. Separable roles for rent1/hUpf1 in altered splicing and decay of nonsense transcripts. *Science* **298**, 419-422 (2002).
9. Consortium., T.G.P. An integrated map of genetic variation from 1,092 human genomes. *Nature (London)* **491**, 56-65 (2012).
10. Wang, Z., *et al.* Systematic identification and analysis of exonic splicing silencers. *Cell* **119**, 831-845 (2004).
11. Zhang, X.H. & Chasin, L.A. Computational definition of sequence motifs governing constitutive exon splicing. *Genes Dev.* **18**, 1241-1250 (2004).
12. Fairbrother, W.G., Yeh, R.F., Sharp, P.A. & Burge, C.B. Predictive identification of exonic splicing enhancers in human genes. *Science* **297**, 1007-1013 (2002).
13. Smith, P.J., *et al.* An increased specificity score matrix for the prediction of SF2/ASF-specific exonic splicing enhancers. *Hum. Mol. Genet.* **15**, 2490-2508 (2006).
14. Zhang, C., Li, W.H., Krainer, A.R. & Zhang, M.Q. RNA landscape of evolution for optimal exon and intron discrimination. *Proc. Natl. Acad. Sci. USA* **105**, 5797-5802 (2008).
15. Divina, P., Kvitkovicova, A. & Vorechovsky, I. *Ab initio* prediction of cryptic splice-site activation and exon skipping. *Eur. J. Hum. Genet.* **17**, 759-765 (2009).
16. Corvelo, A., Hallegger, M., Smith, C.W. & Eyras, E. Genome-wide association between branch point properties and alternative splicing. *PLoS Comput Biol* **6**, e1001016 (2010).
17. Przychodzen, B., *et al.* Patterns of missplicing due to somatic U2AF1 mutations in myeloid neoplasms. *Blood* **122**, 999-1006 (2013).
18. Paz, A., *et al.* SPIKE: a database of highly curated human signaling pathways. *Nucleic Acids Res.* **39**, D793-799 (2011).
19. Lianoglou, S., Garg, V., Yang, J.L., Leslie, C.S. & Mayr, C. Ubiquitously transcribed genes use alternative polyadenylation to achieve tissue-specific expression. *Genes Dev.* **27**, 2380-2396 (2013).
20. Kralovicova, J., Houngninou-Molango, S., Kramer, A. & Vorechovsky, I. Branch sites haplotypes that control alternative splicing. *Hum. Mol. Genet.* **13**, 3189-3202 (2004).
